# Enabling Low Cost Biopharmaceuticals: A Systematic Approach to Delete Proteases from a Well-Known Protein Production Host *Trichoderma reesei*

**DOI:** 10.1371/journal.pone.0134723

**Published:** 2015-08-26

**Authors:** Christopher P. Landowski, Anne Huuskonen, Ramon Wahl, Ann Westerholm-Parvinen, Anne Kanerva, Anna-Liisa Hänninen, Noora Salovuori, Merja Penttilä, Jari Natunen, Christian Ostermeier, Bernhard Helk, Juhani Saarinen, Markku Saloheimo

**Affiliations:** 1 VTT Technical Research Centre of Finland, Espoo, Finland; 2 Novartis Pharma AG, Basel, Switzerland; 3 Glykos Finland Oy, Helsinki, Finland; AIT Austrian Institute of Technology GmbH, AUSTRIA

## Abstract

The filamentous fungus *Trichoderma reesei* has tremendous capability to secrete proteins. Therefore, it would be an excellent host for producing high levels of therapeutic proteins at low cost. Developing a filamentous fungus to produce sensitive therapeutic proteins requires that protease secretion is drastically reduced. We have identified 13 major secreted proteases that are related to degradation of therapeutic antibodies, interferon alpha 2b, and insulin like growth factor. The major proteases observed were aspartic, glutamic, subtilisin-like, and trypsin-like proteases. The seven most problematic proteases were sequentially removed from a strain to develop it for producing therapeutic proteins. After this the protease activity in the supernatant was dramatically reduced down to 4% of the original level based upon a casein substrate. When antibody was incubated in the six protease deletion strain supernatant, the heavy chain remained fully intact and no degradation products were observed. Interferon alpha 2b and insulin like growth factor were less stable in the same supernatant, but full length proteins remained when incubated overnight, in contrast to the original strain. As additional benefits, the multiple protease deletions have led to faster strain growth and higher levels of total protein in the culture supernatant.

## Introduction

The filamentous fungus *Trichoderma reesei* is an efficient producer of extracellular lignocellulose degrading enzymes and is used as a production organism by enzyme industries world-wide. It is amenable to large scale fermentation processes and has a long history of safe use in the enzyme production industry. Several *T*. *reesei* enzymes have obtained the generally recognized as safe (GRAS) status by the U.S. Food and Drug Administration. The protein synthesis and secretion capacity of the fungus is excellent. The highest published amount of extracellular protein produced was over 100 g per liter of culture medium [1]. *T*. *reesei* has tremendous prospects to produce therapeutic proteins in large amounts based upon its secretion abilities. *T*. *reesei* possesses a favorable glycosylation pattern, with around 80% of the N-glycans being of the Man_5_ type [2,3]. Furthermore, *T*. *reesei* is a low cost production system that can be cultivated on inexpensive medium with relatively short cultivation times.

While it is capable of high levels of protein production, *T*. *reesei* is also an active secretor of proteases. This limits the production of many sensitive therapeutic hormones and cytokines that are by nature easy to degrade. Even antibodies which are thought to be relatively stable molecules are susceptible to protease degradation. Only two mammalian proteins have been reported to be produced in *T*. *reesei* [4]. Calf chymosin and a murine Fab fragment were both produced at 150 mg/L when expressed as CBHI-carrier fusions [5,6]. These early production strains had the full complement of secreted proteases making high level production challenging. Higher production levels in *T*. *reesei* have been reported for more stable fungal enzymes such as, *T*. *reesei* tyrosinase at 1 g/L [7] and *Melanocarpus albomyces* laccase at 0.9 g/L [8].

Production of fungal proteases has long been identified as a barrier to achieving high production levels of heterologous proteins [9,10]. In microbial production systems the protease problem has been reduced or overcome by deleting multiple protease genes. Heterologous protein expression was improved using this approach in *Aspergillus oryzae* for lysozyme and chymosin [11–13], *Aspergillus niger* for laccase [14], *Schizosaccharomyces pombe* with human growth hormone [15], and *Ogataea minuta* with antibody [16]. In *A*. *oryzae*, disruption of 10 protease genes was performed by *pyrG* marker recycling in a Δ*ligD* background strain and led to higher yields of bovine chymosin and human lysozyme [17].

Alternatively, particularly when genome sequence information was unavailable, protease deficient strains have been made using classical mutagenesis and screening in *A*. *niger* [18], *T*. *reesei* [19], and *Chrysosporium lucknowense* [20]. One benefit of random mutagenesis approaches is that they may have the capability to achieve wide downregulation of protease gene expression if regulatory genes are mutated in the process. Studying the *A*. *niger* mutant strains led to discovering a unique regulatory factor, PrtT, that controls protease expression in several *Aspergillus* species [9,21]. The *prtT* gene disruptant in *A*. *oryzae* demonstrated lower secretion levels of alkaline serine protease (AlpA) and neutral metalloprotease I (NpI). In *A*. *fumigatus*, the expression of six secreted proteases was significantly reduced upon deletion of *prtT* [22].

With the large number of proteases expressed by *A*. *niger* [23] and *T*. *reesei* [24], for example, it may be impractical to control or delete them all. Thus, one approach to help reduce protease secretion, in parallel to strain improvement, would involve controlling media conditions. Research conducted in *A*. *nidulans* has demonstrated that secreted protease regulation is complex and linked with both carbon and nitrogen regulation [25,26]. The extracellular proteases serve to degrade proteins into smaller units to provide the fungal cells with nutrients, particularly when preferred carbon and nitrogen sources are in short supply. Studies in various fungi have been done to investigate the effect of pH and the various media components upon protease secretion and activity [27–29]. These studies indicated that pH and nitrogen content of the medium can be manipulated in some cases to reduce the secreted protease activity levels.

Given the importance of *T*. *reesei* as large scale producer of cellulases used in many industrial processes, there are surprisingly few studies published concerning the secreted proteases. There have been studies generally describing acid proteases [30,31], a trypsin like serine protease [32], and an alkaline serine protease [33], which were found to affect the stability of the cellulase enzymes [34]. Proteomic studies on *T*. *reesei* QM6a have identified numerous secreted proteases from culture supernatant. There were 39 reported and their expression was described to be pH dependent [35].

There are no previously published reports about specific *T*. *reesei* proteases that are responsible for degradation of therapeutic proteins. Thus, we systematically identified 13 major proteases with inhibitor studies and purification work. The 7 most problematic proteases were successively deleted from the M124 base strain to create a more productive *T*. *reesei* strain suitable for therapeutic protein production. This strain with 7 proteases deleted is sufficient to support stable expression levels of therapeutic proteins, especially antibodies. Therapeutic proteins more susceptible to protease degradation such as interferon alpha 2b (IFNα2b) or insulin like growth factor (IGF1), have greatly benefited from the elimination of proteases, but may require few additional deletions to further increase yields and stabilize the products.

## Materials and Methods

### Chemical list

PMSF (Sigma #P7626), chymostatin (Sigma #C7268), pepstatin (Sigma #P5318), E-64 (Sigma #E3121), EDTA, human IgG (Sigma #I2511). The LIP peptide (AARLARRAGRRSAPFRNDTS-OH) and the SIP peptide (Ac-Phe-Lys-Phe-(3S,4S)-phenylstatinyl-Leu-Arg-NH_2_) were custom synthesized by Almac Sciences (Scotland, UK).

### Strains

*Escherichia coli* DH5α (Life Technologies) was used for propagation of the plasmids. *Trichoderma reesei* QM6a (VTT-D-071262T, ATCC13631), M44, and M124 (M44 Δ*mus53*) were obtained from VTT Culture Collection (Espoo, Finland). Spore suspensions were prepared by cultivating the fungus on potato-dextrose plates (BD, Sparks, Maryland, USA) for 5 days, after which the spores were harvested, suspended in a buffer containing 0.8% NaCl, 0.025% Tween-20 and 20% glycerol, filtered through cotton, and stored at -80°C. *Saccharomyces cerevisiae* strain H3488/FY834 was used to create deletion construct containing plasmid vectors.

### Deletion vectors

Deletion vectors were created for 7 protease genes: *pep1* (tre74156), *tsp1* (tre73897), *slp1* (tre51365), *gap1* (tre69555), *gap2* (tre106661), *pep4* (tre77579), and *pep5* (tre81004). The gene identifiers are listed according to the Joint Genome Institute *T*. *reesei* assembly release version 2.0. The deletion vectors contained the 5′ and 3′ flanking regions of the target gene, a *pyr4* selection marker with a loopout fragment, and the pRS426 vector backbone (S1 Fig).

The 5′ and 3′ flank (1000 bp) fragments, the *pyr4* selection marker, and the 5′ direct repeat fragment (308 bp of *pyr4* 5′UTR) were created via PCR using oligos listed in S1 Table. Template DNA used to amplify these fragments was from the *T*. *reesei* wild type strain QM6a, which is the genome sequenced strain. PCR amplification was performed with Phusion polymerase (Thermo Scientific) or Dynazyme EXT polymerase (Thermo Scientific) with either HF buffer or GC buffer. To prepare the vector backbone pRS426 for cloning it was digested with restriction enzymes *Eco*RI and *Xho*I. All PCR reactions and digestion reactions were separated with agarose gel electrophoresis and isolated with a gel extraction kit (Qiagen).

The purified DNA fragments were transformed into *S*. *cerevisiae* (strain H3488/FY834) to create the final deletion vectors. This homologous recombination based cloning method facilitates vector creation as described in Gietz *et al*. [36]. All DNA fragments to be combined contained 40 base pair overlapping sequences needed for homologous recombination in yeast. The fully assembled plasmid was recovered from yeast, transformed into *E*.*coli*, purified, and checked by restriction digests and sequencing. To release the deletion construct the plasmid was digested with *Pme*I and the correct fragment was purified from an agarose gel using QIAquick Gel Extraction Kit (Qiagen).

The deletion constructs were designed to enable removal of the *pyr4* selection marker from the *T*. *reesei* genome after successful integration and thereby recycling the selection marker for subsequent protease gene deletions. Removal of *pyr4* gene from the deletion construct resembles the so-called blaster cassettes developed for yeasts [37]. Similar deletion constructs have also been developed for filamentous fungi including *Trichoderma* [38].

### Strain generation

Creation of the multiple protease deletion strain began with strain M124, which has a deletion of the *mus53* gene that is important in the nonhomologous end joining (NHEJ) DNA repair pathway. Disrupting the NHEJ pathway improves locus specific integration of DNA and facilitates more efficient strain construction, as we have reported earlier [39]. A *pyr4-* version of M124 was first generated by selection on 5-fluoroorotic acid (5-FOA) plates creating the strain M127 [40]. The strain was transformed with 5 μg of the *pep1* deletion construct and positive transformants were selected for on minimal media containing transformation plates (20 g/L glucose, 15 g/L KH_2_PO_4_, 5 g/L (NH_4_)_2_SO_4_, 182 g/L sorbitol, 18 g/L noble agar, 5 g/L FeSO_4_-7 H_2_O, 1.6 g/L MnSO_4_-H_2_O, 1.4 g/L ZnSO_4_-7 H_2_O, 3.7 g/L CoCl_2_-6 H_2_O, 2.4 mM MgSO_4_, 4.1 mM CaCl_2_, and adjusted to pH 5.5) as described in Gruber *et al*. [40] and Penttilä *et al*. [41].

Transformants were picked and streaked onto minimal media plates with 1 ml/L triton-X100. The streaks were screened by PCR to check for proper locus integration of the deletion construct and absence of the protease gene open reading frame. Transformants that were positive for protease gene deletion were purified to single spore clones. The gene deletion was verified by Southern analyses [42] from DNA extracted with an Easy-DNA kit (Invitrogen) and radiolabeled (^32^P), using the HexaLabel Plus or the DecaLabel Plus kits (Fermentas). Southern digestion schemes were designed using Geneious Pro 5.3.6 software. Southern analyses also verified that the transformant contained only one integrated copy of the deletion construct. The resulting strain was named M181.

To reuse *pyr4* as the selection marker, the *pyr4* marker in the deletion construct within the protease gene locus was removed. The *pyr4* marker was constructed to have the ability to loop-out when put under selection pressure from 5-FOA. Spores were spread onto minimal medium plates containing 20 g/L glucose, 2 g/L proteose peptone, 1 ml/L Triton X-100, 5 mM uridine and 1.5 g/L 5-FOA, pH 4.8. 5-FOA resistant colonies were picked after 5–7 days, suspended in 0.9% NaCl thoroughly by vortexing, and filtrated through a cotton-filled pipette tip. To obtain uninuclear clones, filtrates were spread again onto the 5-FOA containing minimal plates. Purified clones derived from a single colony were sporulated on plates containing 39 g/L potato dextrose agarose. These clones were tested for uridine auxotrophy by plating spores onto glucose/triton plates (20 g/L glucose, 1 ml/L Triton X-100, TrMM with and without uridine). Clones growing only in the presence of uridine were further tested by PCR to verify that the *pyr4* marker was removed from the *pep1* locus. Once the strain was *pyr4*- it was again ready for the next protease deletion.

We next proceeded to delete the *tsp1* protease gene following the same process. This same transformation, selection, verification, and marker removal cycle was carried out until the final 7^th^ protease gene was deleted. The final deletion strains were named: M181 (Δ1), M219 (Δ2), M277 (Δ3), M307 (Δ4), M369 (Δ5), M396 (Δ6), and M486 (Δ7). These strains and their parental strains are listed and described in Table 1.

**Table 1:** Strain list.

Strain #	Gene deletions	Previous strain	Description
QM6a	-	-	Original sequenced strain. The genomic DNA was used as PCR template to clone deletion constructs.
M44	-	-	Our production strain. Used to produce the culture supernatant for protease purification.
M124	*mus53*	M44	*Mus53* ligase gene deleted to improve homologous recombination needed for more efficient strain construction. The deletion series started with this strain.
M127	-	M124	*Pyr4*- strain
M169	*mus53*	M44	*Mus53* deletion strain
M181	*pep1*	M124 *pyr4*-	Δ1 protease
M182	*pep1*	M169 *pyr4*-	Δ1 protease
M219	*pep1*, *tsp1*	M181 *pyr4*-	Δ2 proteases
M277	*pep1*, *tsp1*, *slp1*	M219 *pyr4*-	Δ3 proteases
M307	*pep1*, *tsp1*, *slp1*, *gap1*	M277 *pyr4*-	Δ4 proteases
M369	*pep1*, *tsp1*, *slp1*, *gap1*, *gap2*	M307 *pyr4*-	Δ5 proteases
M396	*pep1*, *tsp1*, *slp1*, *gap1*, *gap2*, *pep4*	M369 *pyr4*-	Δ6 proteases
M486	*pep1*, *tsp1*, *slp1*, *gap1*, *gap2*, *pep4*, *pep3*	M396 *pyr4*-	Δ7 proteases

### Fermentor cultivations

For analysis of protease content in the supernatant, the M44 strain was cultivated in 10 L fermentor in *Trichoderma* minimal medium (TrMM) supplemented with 20 g/L spent grain extract and 60 g/L lactose with lactose feeding at pH 5.5 and at 28°C. The lactose feeding was controlled via the control paradigm DELTABAS, as described in Bailey *et al*. [43]. The fed batch cultivation was continued for 9 days. TrMM contains 7.6 g/L (NH_4_)_2_SO_4_, 15.0 g/L KH_2_PO_4_, 2.4 mM MgSO_4_-7H_2_O, 4.1 mM CaCI_2_-H_2_O, 3.7 mg/L CoCI_2_, 5 mg/L FeSO_4_-7H_2_O, 1.4 mg/L ZnSO_4_-7H_2_O and 1.6 mg/L MnSO_4_-7H_2_O [41]. M277 was cultivated in TrMM medium supplemented with 20 g/L spent grain extract, 60 g/L lactose, and 9 g/L casamino acids at pH 5.5 and 28°C. The M182 and M169 were cultivated in fermentor in TrMM medium supplemented with 20 g/L spent grain extract, 60 g/L lactose, 8.1 g/L casamino acids at pH 5.5 and 28°C.

For analysis of the protease deletion strain performance, all deletion strains were cultivated in 1L batch fermentations using a 2L DASGIP Parallel Bioreactor System. Cultivations have been carried out in cellulase-inducing medium TrMM plus 20 g/L yeast extract, 40 g/L cellulose, 40 g/L cellobiose, 20 g/L sorbose, at pH 5.5 and 28°C for 3 days. The biomass capacitance in all reactors was on-line monitored using ABER Futura probes (Aber Instruments, Wales, UK).

### Shake flask cultures

Shake flask culture supernatants were occasionally used for assaying proteolytic activity against model proteins when fermentation supernatant was not available. The cultures were started with 6 x10^7^ spores/300 ml medium and grown in 2 liter flasks with 300 ml TrMM containing 40 g/L lactose, 20 g/L spent grain extract, and 100 mM PIPPS at pH 5.5 or pH 4.5 at 28°C and shaking at 200 rpm.

### Protease identification with inhibitors

The degradation of human IgG (Sigma #I2511) was explored with and without different protease inhibitors in the supernatant from the fed batch fermentation performed with strain M44 at pH 5.5 and 28°C. The culture supernatant was diluted with sodium citrate buffer (50 mM, pH 5.5) to total protein level of 12 mg/ml. The original total protein concentration was 23.4 mg/ml. Antibody (50 μg/ml) was added to the diluted supernatant in the presence and absence of pepstatin A (100 μM), E-64 (20 μM), PMSF (10 mM), and EDTA (10 mM). The samples were taken after 0 and 19 hours of incubation at 37°C and the nonreduced IgG samples were analyzed via SDS PAGE analysis using low range prestained molecular weight markers (BioRad).

### LC-MS/MS analysis and sample preparation

The desired bands were excised from the stained gel with a razor blade. The gel pieces were cut into 0.5 x 0.5 mm cubes and put into an Eppendorf tube. The Coomassie stained gel pieces were destained with three changes of 200 μl of 0.25 M NH_4_HCO_3_/ACN (1:1) for 20 minutes at 37°C. The gel pieces were shrunk by twice adding 100–200 μl ACN until pieces became white. The pieces were dried in the vacuum centrifuge for 5 minutes. After that the pieces were rehydrated in 100 μl of 20 mM DTT 0.1 M NH_4_HCO_3_ for 30 minutes at 56°C. The excess liquid was removed and pieces shrunk with ACN. To the dried pieces 100 μl of 55 mM iodoacetamide in 0.1 M NH_4_HCO_3_ was added and incubated in the dark at room temperature for 15 minutes. The liquid was removed around the gel pieces and washed with 200 μl of NH_4_HCO_3_ and shrunk with ACN. To the dried gel pieces modified trypsin (0.05 μg/μl, Promega #V5111) was added and allowed to absorb for 30 minutes at room temperature. Digestion buffer (0.1 M NH_4_HCO_3_/10% ACN) was added to wet the gel pieces and incubated overnight at 37°C. The supernatant was recovered and the gel pieces were incubated twice in 10 μl of 5% formic acid. The supernatants were combined and prepared for peptide purification. The tryptic peptides were purified using a ZipTip (Millipore C18 #ZTC18M096) according to manufacturer’s protocol and eluted using 75% ACN. The dried purified peptides were analyzed by LC-MS/MS analysis on a QSTAR Pulsar, ESI-hybrid quadrupole-TOF (AB Sciex) at the Turku Biotechnology Center Proteomics Facility.

### Total protein assays

Total protein measurements were done with the Bradford assay reagent (Coomassie Plus Thermo #23238) according to the manufacturer’s instructions with bovine immunoglobulin as a standard. Typically the fermentation supernatants were diluted 1:30 or 1:40 in sodium citrate buffer and compared to the standard curve and measured with absorbance at 595 nm.

### Protease assays with casein

Protease activity against casein was tested using the EnzChek protease assay kit (Molecular probes #E6638, green fluorescent casein substrate) or the QuantiCleave protease assay kit (Pierce #23263, succinylated casein). The working stock solution was prepared by diluting the stock to 10 μg/ml in 50 mM sodium citrate, pH 4.5 or pH 5.5. The purified protease fractions were diluted with sodium citrate buffer. 100 μl of the diluted substrate was combined with the diluted protease fractions in a 96 well sample plate. The plate was then covered and kept at 37°C for one to three hours. Fluorescence readings were taken at one, two, and three hours with a Varioskan fluorescent plate reader (Thermo Scientific) using 485 nm excitation and 530 nm emission. To measure the QuantiCleave assay reaction 50 μl of TNBSA reagent was added to every well and the plate was incubated at 37°C. The absorbance at 450 nm was measured for the whole plate. Control wells with supernatant without substrate were used as background controls. The nonspecific background signal was subtracted from specific protease activity measurement.

### Aspartic protease purification and identification

Aspartic proteases were affinity purified from the M44 culture supernatant using pepstatin A attached to agarose (Sigma #P2032). The supernatant from 9 days of cultivation contained 23 g/L total protein. The supernatant (15 ml) was batch bound to the resin in 35 ml buffer containing 50 mM sodium acetate and 0.2 M NaCl at pH 3.0. The column was washed with the same binding buffer and bound protein was removed with elution buffer (50 mM Tris-HCL, 1 M NaCl, pH 8.5). Fractions of 0.5 ml were collected. 30 μl of each fraction was mixed with 6 μl of Laemmli sample buffer containing β-mercaptoethanol. The samples were heated at 95°C for 5 minutes before being loaded into a 4–15% PAGE gel (BioRad mini-protean TGX precast gel) along with a low range prestained molecular weight marker (BioRad). The gel was run in SDS PAGE running buffer for 30 minutes at 100 V and then stained with GelCode blue stain (Thermo Scientific).

The 42 kDa doublet band was excised from the SDS PAGE gel and subjected to in-gel trypsin digestion with sequencing grade modified trypsin (Promega #V5111). The resulting peptides were then extracted from the gel and purified by C18 ZipTip (Millipore #ZTC18M096). The purified peptides were analyzed by LC-MS/MS on a QSTAR Pulsar, ESI-hybrid quadrupole-TOF (AB Sciex).

### Aspartic protease activity studies with immunoglobulin

Protein (0.8 μg) from the peak fraction (F3) with and without pepstatin A (100 μM) was incubated with IgG (50 μg/ml) in sodium citrate buffer (50 mM, pH 5.5) at 37°C for 20 hours. Samples were taken at 0, 1 hour, and 20 hours and analyzed by SDS-PAGE gel and immunoblotting.

The protein was incubated either in the presence or absence of 10 μM pepstatin A. The antibody mixture was combined with Laemmli sample buffer and heated at 95°C for 5 minutes. These samples were then loaded into a 4–15% PAGE gel (BioRad mini-protean TGX precast gel) along with a broad range prestained molecular weight marker (BioRad) and run for 30 minutes at 100 V. The IgG was not reduced before being run on the gel. Full size IgG runs just above the 100 kDa marker.

### Analysis of *pep1* deletion strains

The M182 Δ*pep1* strain and the parental control strain M169 were cultivated in fermentor as described above. Aspartic proteases were purified using a pepstatin-agarose affinity column from 15 ml of supernatant and analyzed in a 12% SDS PAGE gel with low range molecular weight markers.

Shake flask cultures were grown with a second set of strains M181 Δ*pep1* and the parental strain M124. The cultures were grown in TrMM plus 20 g/L spent grain extract, 40 g/L lactose, and 100 mM PIPPS at pH 5.5 and 28°C. The shake flask supernatants from day 5 and control fed batch supernatant from M44 were diluted to 2 mg/L total protein and incubated with model antibody (0.05 μg/μl final concentration) overnight for 18 hours at 37°C. Three different model antibodies studied were rituximab, MAB01, and MAB02.

Supernatant samples (30 μl) containing antibody were combined with Laemmli sample buffer, heated at 95°C for 5 minutes, and loaded into a 4–15% SDS PAGE gel (BioRad mini-protean TGX precast gel) along with high range prestained molecular weight markers (BioRad). The gel was run in SDS PAGE running buffer for 30 minutes at 200 V. The proteins in the gel were electrotransferred into a nitrocellulose filter at 100 V for 1 hour. The protein containing nitrocellulose filter was then blocked with 5% milk powder in TBST for 1 hour shaking at room temperature. The blocked membrane was then probed with antibody. The nitrocellulose membrane was incubated with anti-heavy chain (Sigma #A3188) and anti-light chain (Sigma #A3813) AP conjugates diluted 1:30,000 in TBST. After 1 hour incubation with detection antibody, the membrane was washed with TBST for 1 hour. The membrane was developed with the BCIP/NBT alkaline phosphatase substrate (Promega #S3771) for up to 5 minutes.

### SIP affinity purification

Several additional aspartic proteases were isolated from the M277 (Δ3) strain fermentor culture supernatant. The proteases were isolated by affinity purification using the SIP peptide (Ac-Phe-Lys-Phe-(AHPPA)-Leu-Arg-NH_2_), which is known to bind glutamic proteases [44]. The SIP peptide was conjugated to NHS activated agarose resin (Thermo Scientific #26196) using the protocol provided by the manufacturer. Fermentation supernatant (15 ml) was used to batch bind proteases to the resin in 35 ml buffer containing 50 mM sodium acetate, 0.2 M NaCl, pH 3.0. The column was washed with the same binding buffer and bound protein was removed with elution buffer (50 mM Tris-HCL, 1 M NaCl, pH 8.5). Fractions of 0.5 ml were then collected.

30 μl of each purified fraction was then run on a 4–15% SDS PAGE gel (BioRad mini-protean TGX precast gel) and stained overnight with GelCode blue (Thermo Scientific #24590). The bands from the gel were then cut out and subjected to in-gel trypsin digestion with sequencing grade modified trypsin (Promega #V5111). The resulting peptides were extracted from the gel and purified by C18 ZipTip (Millipore #ZTC18M096). The purified peptides were analyzed by LC-MS/MS on a QSTAR Pulsar, ESI-hybrid quadrupole-TOF (AB Sciex).

### Serine protease inhibitor studies

The serine protease inhibitors chymostatin and SBTI were analyzed *in vitro* with culture supernatant. Supernatant from the M44 fed batch fermentor culture sampled after 9 days was diluted to 6 mg/ml with sodium citrate buffer pH 5.5. To this diluted supernatant 0.05 μg/μl of rituximab, 100 μM chymostatin, 1 mg/ml SBTI, or a combination of both inhibitors was added in a total volume of 50 μl. Reactions were incubated at 37°C and sampled at 0, 1, and 19 hours to assess the early and late degradation of the rituximab antibody heavy chain. The resulting heavy chain products were run in a 12% SDS PAGE gel and analyzed by immunoblot using and anti-heavy chain AP conjugated antibody (Sigma #A3188) diluted 1:30,000 in TBST.

### Serine protease purification and activity

Benzamidine sepharose 4 fast flow resin (GE healthcare # 17-5123-10) was used to purify serine protease enzymes from the M44 culture supernatant sampled after 9 days. 15 ml of the fermentation culture supernatant was batch bound to the resin in 35 ml of binding buffer (0.05 M Tris-HCL, 0.5 M NaCl, pH 7.4). After packing and washing the column with the same binding buffer, the column was eluted with 0.05 M glycine, pH 3.0. The fractions were then neutralized with 1M Tris HCL, pH 8.8. The peak fraction protein mixture (F4) and IgG were incubated together with and without PMSF in sodium citrate buffer (50 mM, pH 5.5) at 37°C for 20 hours. The nonreduced samples were loaded into a 12% SDS PAGE gel and an immunoblot with heavy and light chain AP conjugated antibodies was done to analyze the reaction samples.

### SBTI and chymostatin affinity purification

A 20 ml sample of M44 culture supernatant from a 9 day sample was incubated with the SBTI-agarose affinity resin (Sigma #T0637; 1 ml) in 30 ml of binding buffer (50 mM Tris, 0.5 M NaCl, pH 7.5). The supernatant binding buffer mixture was combined in a 50 ml conical tube and agitated at room temperature for 1 hour. The mixture was then added to a glass column and washed with 200 ml of binding buffer. 50 ml of high salt buffer (1 M NaCl) was next used to further remove nonspecific interactions. Finally, the column was washed again with 100 ml of the original binding/wash buffer. The column was then eluted with 0.8 M benzamidine HCl in 50 mM Tris, pH 5.0. The fractions were collected in 0.5 ml volumes and subjected to a protein assay using BioRad Bradford reagent with bovine immunoglobulin as a standard. The peak fraction was washed in a Vivaspin ultrafiltration spin filter (Sartorius-stedim) with 10 kDa molecular weight cutoff to remove the benzamidine inhibitor and concentrate the fraction.

To analyze the proteolytic activity of the purified protease of concentrated fraction #3, the fraction was tested for its ability to degrade the rituximab antibody heavy chain. A 5 μl sample of concentrated fraction #3 was incubated in sodium citrate buffer pH 5.5 with 0.05 μg/ml rituximab at 37°C for 18 hours. The incubated samples were then analyzed by immunoblotting using an anti-human IgG heavy chain-specific AP conjugated antibody (Sigma #A3188) diluted 1:30,000 in TBST.

The chymostatin affinity column was generated by coupling chymostatin to the carboxylink DADPA activated agarose resin (Thermo Scientific #44899) according to the manufacturer’s protocol. Proteases were purified from the M277 (Δ3) strain according to the same procedure described above for SBTI. Purified fractions were digested with trypsin, peptides were isolated, and analyzed by LC-MS/MS.

### Zymogram analysis

In order to identify which proteins in the purified fractions exhibited protease activity, the fractions were run on an IgG zymogram gel. Zymogram gels were cast with immunoglobulin (0.5 mg/ml MAB02) in a normal 12% SDS PAGE gel. The purified fractions and unpurified supernatant samples were run on the zymogram gel under denaturing conditions. After running the gel, the proteins in the gel were renatured by incubating the gel in 2.5% Triton X-100 for one hour to remove the SDS. The zymogram gel was then allowed to incubate overnight in reaction buffer (50 mM sodium citrate, pH 5.5) so that the proteases could degrade IgG in the gel. The gel was then stained with GelCode blue (Thermo Scientific) to reveal the extent of IgG staining. Active proteases produced a clear band with no staining.

The proteases responsible for the zymogram activities were identified by LC-MS/MS peptide sequencing. Protein containing gel sections were cut out of the matched normal SDS PAGE gels and subjected to in-gel trypsin digestion with sequencing grade modified trypsin (Promega #V5111). The resulting peptides were extracted from the gel and purified by C18 ZipTip (Millipore #ZTC18M096). The purified peptides were analyzed by LC-MS/MS on a QSTAR Pulsar, ESI-hybrid quadrupole-TOF (AB Sciex).

### Degradation studies with 3-fold and 6-fold protease deletion supernatants

The supernatant from M277 (Δ3) and M124 shake flask cultures (day 5 and 7) was diluted so that the protein concentration was 6 mg/ml in 50 mM sodium citrate buffer. To these diluted supernatants the MAB01 antibody was spiked into a final concentration of 0.05 μg/μl. These reactions were incubated at 37°C for 18 hours. The reactions were sampled at zero time, 1 hour, and overnight incubation. The 20 μl samples were loaded into a 4–15% SDS PAGE gel and run at 200 V for 40 minutes. The gel was transferred to nitrocellulose at 100 V for 1 hour for immunoblotting. The membrane was blocked with 5% milk in TBST for one hour. The heavy chain of MAB01 was detected with an anti-heavy chain AP conjugated antibody (Sigma #A3188) diluted 1:30,000 in TBST. After washing the membrane with TBST, the blot was developed with AP substrate (Promega).

The protease stability of the model proteins IGF, IFNα2b, and MAB01 were analyzed by spiking them into supernatant from the M396 (Δ6) strain. The supernatant was collected from a large shake flask culture. The undiluted supernatant from the shake flask cultivation was incubated with the purified model proteins with and without pepstatin A (50 μM) and SBTI (0.2 mg/ml) inhibitors for 20 hours at 37°C. The 5 day culture supernatant pH was around 4.2. The reaction containing 0.05 μg/μl of model protein was sampled after 20 hours. 50 mM sodium citrate pH 4.0 spiked with model proteins (0.05 μg/μl) was used as a buffer control.

From each reaction 10 μl was loaded into an 18% SDS PAGE gel and run for 30 minutes at 200 V. The proteins in the gel were then transferred to nitrocellulose for immunoblotting. The nitrocellulose membrane was blocked for 1 hour at room temperature with 5% milk in TBST buffer. The individual blots were probed with their specific primary antibody to detect the appropriate model protein for 1 hour at room temperature on a shaker. The mouse anti-IGF1 antibody (R&D systems #mab291) was used at 2 μg/ml diluted in TBST. The mouse anti-rhGH antibody (Abcam #ab51232) was used at 2 μg/ml diluted in TBST. The mouse anti-IFNα2b antibody (Abcam #ab9386) was used at 1 μg/ml diluted in TBST. After briefly washing the blot membranes with TBST, the secondary antibody was added for 1 hour at room temperature with shaking. The secondary goat anti-mouse AP conjugated antibody (Biorad #170–6520) was diluted 1:10,000 in TBST. MAB01 samples were immunoblotted with an anti-heavy chain AP conjugated antibody (Sigma #A3188).

## Results

### Protease activity against human IgG

In order to begin to identify proteases harmful for antibody production in *T*. *reesei*, the secreted proteases of this fungus at pH 5.5 were characterized using different protease inhibitors. Fermentor culture supernatant from strain M44 was used to degrade human IgG in the presence and absence of protease inhibitors. The antibody analyzed was not reduced so it would run as the 150 kDa assembled form in the gel. PMSF, a serine protease inhibitor, was able to inhibit half of the degradation caused by *T*. *reesei* proteases. The full length IgG band and one lower band were visible after incubation with PMSF treated supernatant. The pepstatin A treatment only slightly repressed the degradation of immunoglobulin. There was no full length IgG; however a smaller digestion product was still visible after treatment. E-64, cysteine protease inhibitor, and EDTA, metalloprotease inhibitor, were unable to inhibit the degradation of the immunoglobulin. The buffer control was perfectly stable after incubation for 19 hours. The 0 time point taken from the untreated supernatant sample also showed full sized antibody. Thus, the data would suggest that both serine and aspartic proteases have major roles in proteolyzing the IgG, but the serine proteases reacting to PMSF were of greater importance under pH 5.5 conditions.

### Aspartic proteases

It was found that part of the protease activity in culture supernatants could be inhibited with the aspartic protease inhibitor pepstatin A. To identify the specific aspartic proteases contributing to the observed protease activity, they were purified from the M44 fermentation supernatant using a pepstatin A affinity column. The aspartic proteases bind the inhibitor peptide and can subsequently be released from the immobilized inhibitor, thus isolating proteases from the supernatant. The eluted protease containing fractions were run onto a PAGE gel for size analysis and the total protein was measured from each fraction. In total 42 μg of protein was purified from the 15 ml of supernatant taken from the fermentation sample. The peak fraction contained 0.04 μg/μl protein. The total protein concentration of the supernatant sample was measured to be 23.4 mg/ml. Aspartic proteases, which bind to pepstatin A peptide, thus make up only 0.012% of the total protein secreted. The bulk of the secreted proteins are cellulase enzymes.

**Fig 1:**
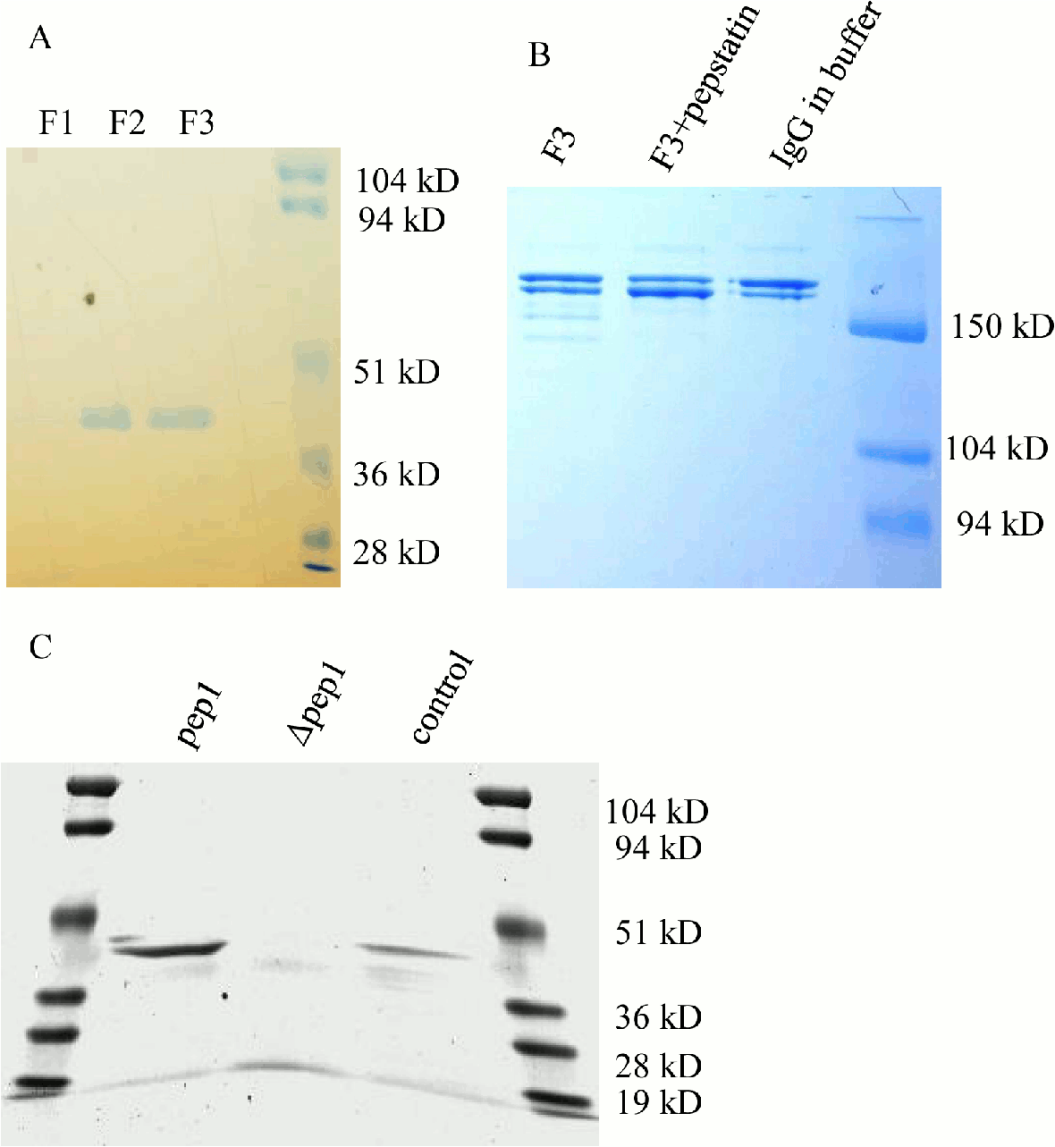
Aspartic protease purification and activity. Fig 1A. Coomassie stained PAGE gel showing fractions eluted from pepstatin affinity purification from the M44 strain. The purified aspartic proteases run around 42 kDa. Fraction numbers are listed above the lanes. Fig 1B. Commassie stained PAGE gel showing aspartic protease degradation of human IgG with and without pepstatin A inhibitor (100 μM). Purified fraction #3 (F3) from the M44 strain was incubated with IgG in sodium citrate buffer at pH 5.5 for 20 hours at 37°C. Nonreduced gel showing the whole assembled antibody and degradation products. The fully assembled antibody runs above 150 kDa. Fig 1C. Commassie stained PAGE gel visualizing the peak fractions from aspartic protease purifications done on the M169 parental strain and M182 Δ*pep1* strain. The control aspartic protease sample was from a previous purification. The predominant band at 42 kDa corresponds to *pep1*.

From the peak fractions a 42 kDa doublet band was observed (Fig 1A) and identified using LC-MS/MS. The mass analysis of peptides from this doublet band identified 4 aspartic proteases including PEP1 (tre74156; 42.7 kDa, 42% sequence coverage), PEP2 (tre53961; 42.4 kDa, 15% sequence coverage), PEP3 (tre121133; 49 kDa, 6% sequence coverage), and PEP5 (tre81004; 45 kDa, 9% sequence coverage). These proteases are between 51%-64% similar in amino acid sequence. When the peak fraction of the purified aspartic proteases was incubated with human IgG at pH 5.5 it produced degradation products that were inhibitable by pepstatin A treatment (Fig 1B). The buffer control remained intact after 20 hours incubation. Notably this was not an optimal pH for aspartic proteases as they typically work much better in more acidic conditions. It was clear that aspartic proteases would play a role in antibody degradation under pH 5.5 culture conditions, but their effects on antibody are mild under these conditions.

The *pep1* protease was by far the most abundant aspartic protease and therefore deleted first in our multiple deletion strain. This was followed by identifying and deleting major serine proteases which appeared to play a larger role, compared to the aspartic proteases, in antibody degradation.

### Purification of aspartic proteases in *pep1* deletion background

Due to it being the most abundant and well known of the aspartic proteases, *pep1* (tre74156) was deleted from the M127 and M169 strains. The *pep1* deletion strain M182 and the parental strain M169 were cultivated in batch fermentation. The aspartic proteases from the M182 Δ*pep1* strain supernatant were similarly purified and compared to the aspartic proteases from the parental M169 strain. From 15 ml of culture supernatant 7 μg of aspartic protease was recovered from the M182 Δ*pep1* strain. When the purified fraction was run on SDS PAGE, the 42 kDa molecular weight band previously seen in the parental strain had mostly disappeared and a faint band around 40 kDa could be seen (Fig 1C). From 15 ml of the parental strain supernatant 17 μg of aspartic protease was purified and a 42 kDa protein was visible on the SDS PAGE gel. According to this analysis, 10 μg of PEP1 protease would be produced per 15 ml of culture supernatant. Thus, PEP1 protease would make up 60% of the total aspartic protease and only 0.008% of total protein in the supernatant. In these batch cultivations the total protein concentration were measured to be 8 mg/ml on the final day. This data demonstrates that PEP1 is the most abundant aspartic protease secreted by *T*. *reesei* under these conditions.

**Fig 2:**
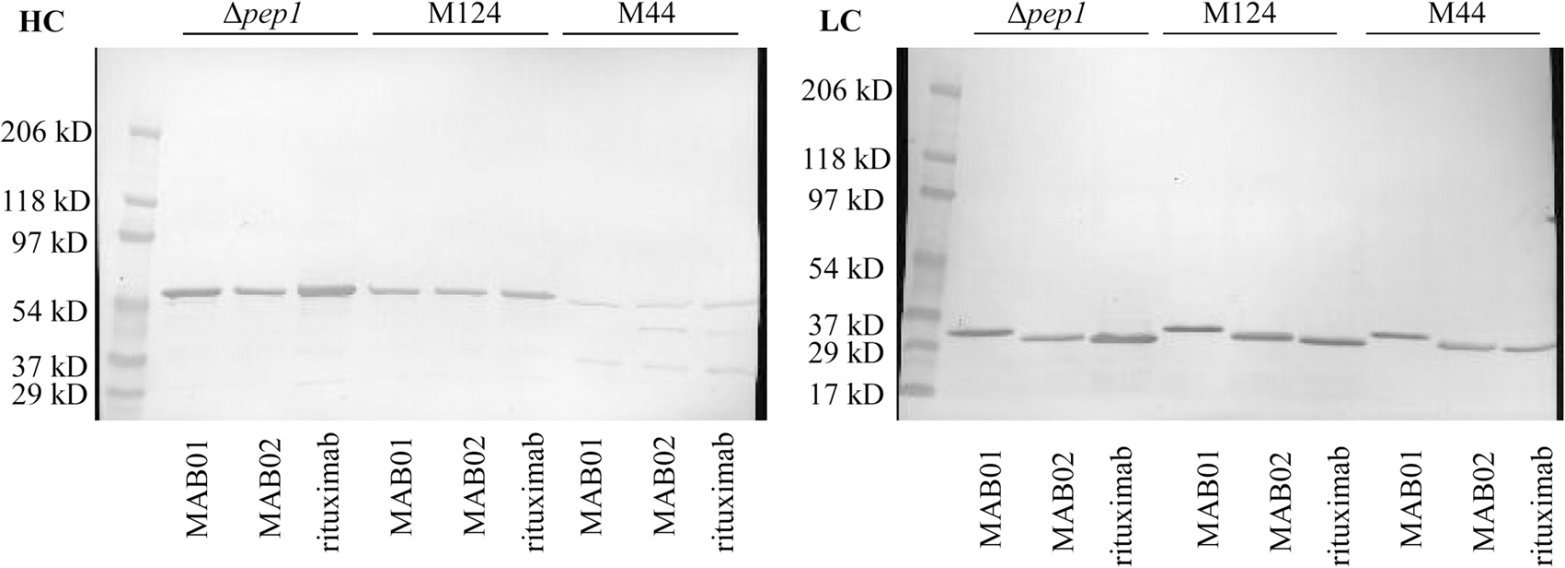
Antibody stability in culture supernatant. Immunoblot showing improved stability of antibody heavy (HC) and light chain (LC). Three model antibodies were tested in large shake flask supernatant (M181 Δ*pep1* and M124) and M44 fermentation supernatant. The supernatants were each diluted so that the total protein was 2 mg/ml for each culture and incubated with antibody for 18 hours at 37°C and pH 5.5.

The shake flask supernatants from the M181 Δ*pep1* strain and the parental strain M124 were used *in vitro* to compare the stability of three antibody molecules. When the shake flask supernatants were incubated with antibody for 18 hours at 37°C and pH 5.5, the Δ*pep1* supernatant degraded less of the heavy chain protein as compared to the control strain (Fig 2). The biggest stabilization effect was evident for rituximab and MAB01 heavy chains. There was only a slight improvement in the stability of light chain protein in the Δ*pep1* supernatant as compared to controls. The protease activity present in the M44 fermentation supernatant was far above that seen with the shake flask supernatants. The diluted M44 fed batch fermentation supernatant was used as a very protease active control. The heavy chain degradation products can be best seen with this treatment. In the heavy chain, two distinct degradation products were seen at ~38 kDa and ~48 kDa. The antibody heavy chain was almost fully degraded when incubated in diluted fermentation supernatant. In comparison the light chain was far more protease resistant than the heavy chain.

### Purification of proteases with SIP peptide

The other major acidic proteases beyond the aspartic proteases were thought to be glutamic proteases. Glutamic proteases do not bind pepstatin A, therefore the SIP peptide was used to capture glutamic proteases [44]. Affinity purification was done with the SIP peptide to attempt to purify glutamic proteases from the M277 (Δ3) fermentation supernatant. The M277 (Δ3) strain was created after identification and deletion of serine proteases reported in the later sections describing serine protease identification. With the three most important proteases removed from the strain, we searched for glutamic proteases. When the purified fractions were run on the SDS PAGE gel, predominant bands at 42 kDa and a minor band around 25 kDa could be seen. The bands from this gel were cut out and digested by trypsin. The trypsin digested peptides were isolated and analyzed by LC-MS/MS. This analysis revealed that the aspartic proteases PEP2 (tre53961), PEP3 (tre121133), PEP4 (tre77579), and PEP5 (tre81004) were present along with the glutamic protease GAP1 (tre69555) and the subtilisin protease SLP2 (tre123244). The aspartic proteases and subtilisin-like protease were found in the 42 kDa area. The glutamic protease was found in the region of the faint 25 kDa band. The GAP1 protease was found to have a sequence homolog, which was called GAP2 (tre106661). Both glutamic proteases were selected for deletion from the multiple deletion strain. Two other sequence homologs exist (tre57575 and tre70927), but their low mRNA expression levels made them less interesting for deletion compared to other candidate proteases.

### Serine protease activity

**Fig 3:**
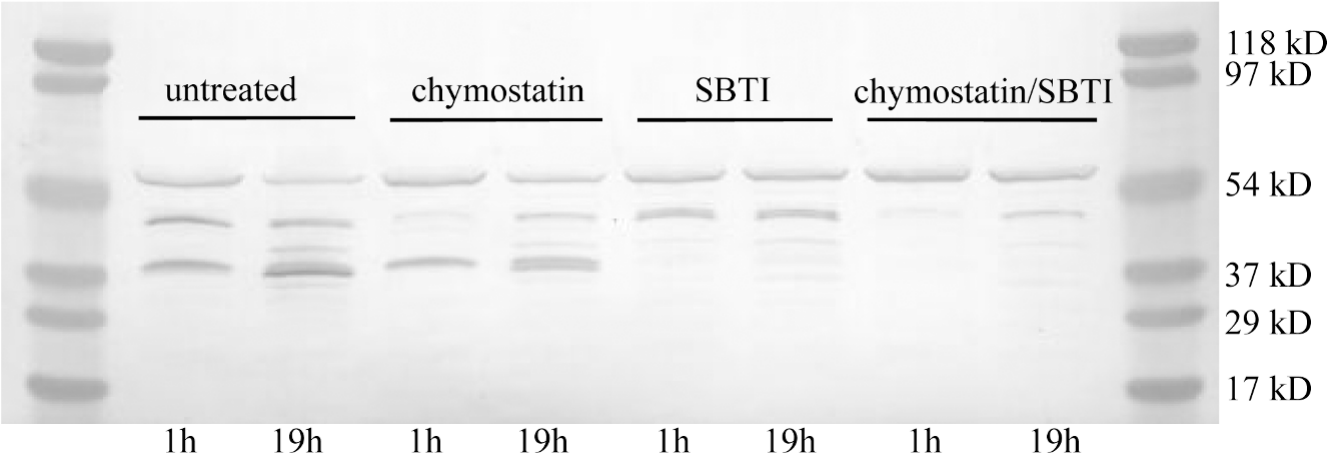
Inhibitor treatments stabilized antibody heavy chain. Immunoblot showing the rituximab heavy chain fragments created by M44 supernatant proteases. The fed batch supernatant was diluted to 6 mg/ml in sodium citrate buffer pH 5.5 and incubated with 0.05 μg/μl of rituximab, 100 μM chymostatin, 1 mg/ml SBTI, or a combination of both inhibitors at 37°C for up to 19 hours.

Based on the knowledge that human IgG was cleaved by serine proteases, tryptic and chymotrypic inhibitors were tested to see if they could prevent rituximab degradation. Chymostatin and SBTI were analyzed *in vitro* with M44 fed batch culture supernatant to check which stabilize the heavy chain of rituximab. The initial degradation products generated after 1 hour from the heavy chain were approximately 38 kDa and 42 kDa, which were seen in the untreated control lane at 1 hour (Fig 3). Additional fragments were generated after 19 hours, but the two major products remained. Chymostatin treatment inhibited the initial production of the 42 kDa fragment, while SBTI treatment inhibited the 38 kDa fragment from forming. Combining the two inhibitors suppressed about 96% of the initial heavy chain degradation and about 75% after 19 hours (Fig 3), measured as a percentage of full length heavy chain signal compared to the degradation products generated. These results demonstrate that the two inhibitors were able to effectively stabilize the rituximab antibody heavy chain. At least when assayed at pH 5.5, serine proteases appear to be the predominant player in terms of heavy chain degradation.

### Serine protease purification and activity studies

**Fig 4:**
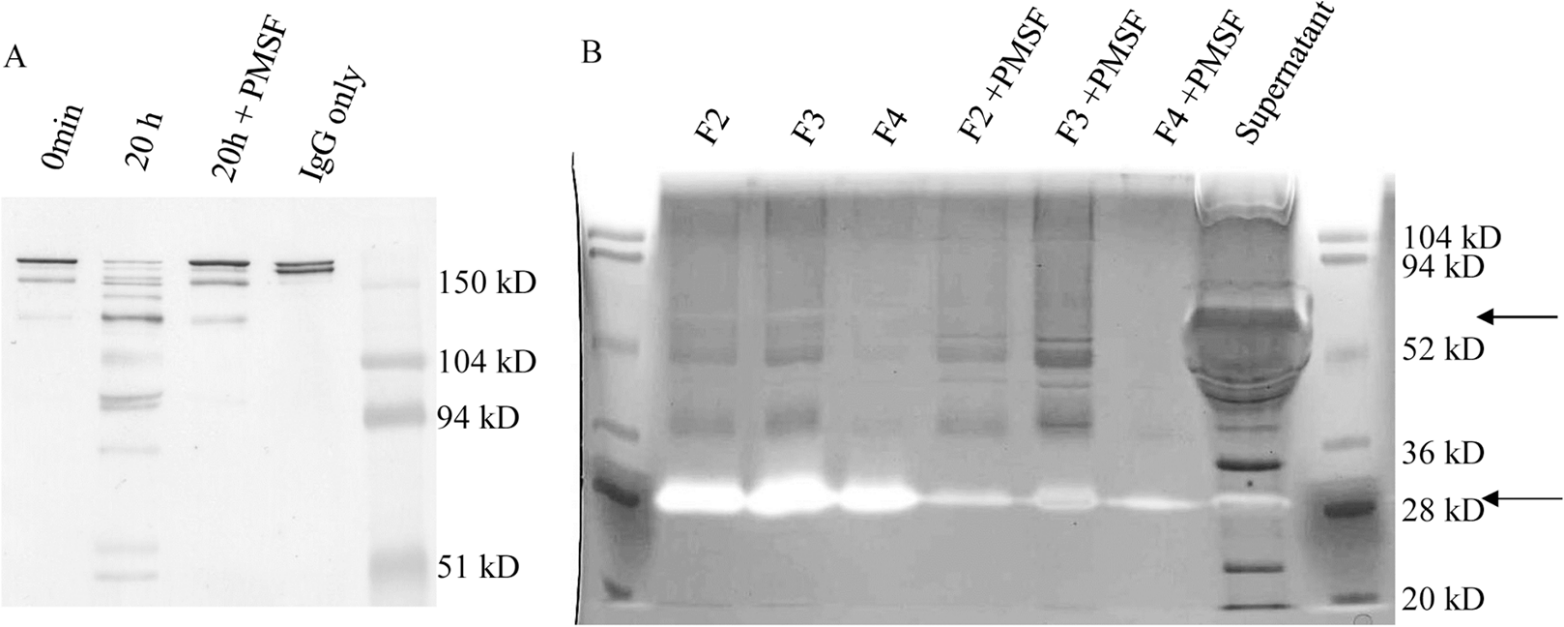
Aminobenzamidine purified serine proteases degraded antibody. Fig 4A. Proteases from the aminobenzamidine affinity purification fraction #4 degraded human IgG. Samples were taken at 0 min and 20 hours after incubating at pH 5.5 and 37°C. The IgG control was antibody in buffer incubated for 20 hours. Immunoblotting was done to detect the heavy and light chain of the nonreduced IgG. The full length antibody runs around 150 kDa. Fig 4B. Zymogram analysis of MAB02 antibody protease degradation from aminobenzamidine column purified fractions (F2-F4) and from M44 supernatant samples. Purified samples were incubated with and without PMSF serine protease inhibitor at pH 5.5. White band indicate degradation of MAB02 antibody. Protease activity came from 65 and 29 kDa, as the arrows indicate.

Serine proteases were purified from the M44 fermentation supernatant using a *p*-aminobenzamidine affinity column. Aminobenzamidine is thought to inhibit trypsin-like serine proteases. From 15 ml of supernatant a total of 1.7 mg of serine protease was recovered from the column. The total serine protease purified represents 0.5% of all the total protein in the supernatant. When the peak fractions were run on a 4–15% SDS-PAGE gel, several major bands (~110 kDa, 53 kDa, 39 kDa, 29 kDa) and many more minor bands were visible. The peak fraction protein mixture (F4) and IgG were incubated together with and without PMSF in sodium citrate buffer (50 mM, pH 5.5) at 37°C for 20 hours. An immunoblot was done on nonreduced antibody to analyze the resulting reaction samples. The purified protease thoroughly degraded the IgG after 20 hours (Fig 4A). Treatment with 5 mM PMSF was able to inhibit most of the degradation, indicating that serine protease enzymes were primarily responsible.

To identify which proteins in the purified fractions were exhibiting protease activity, the peak fractions (F2-F4) were treated with and without PMSF and then run into an IgG (0.5 mg/ml MAB02) zymogram gel (12%). When the purified fractions and unpurified supernatant were analyzed via antibody zymogram there were two clear bands visible on the gel at around 29 kDa and 65 kDa indicating that the IgG in the gel had been degraded (Fig 4B). These bands were also seen in the supernatant control lane, but with less intensity. In the purified fractions the band at 29 kDa was much more predominant, suggesting it may be responsible for most of the serine protease activity in the sample. When the protein sample was pretreated with PMSF, these clear white bands appeared grey or were not visible, indicating that they were serine protease enzymes. These 65 kDa and 29 kDa bands were visible in the unpurified supernatant sample. There were also some unidentified zymogram activity bands seen in the supernatant above 104 kDa. When the zymogram studies were conducted at pH 5.5 the protease activities could be clearly seen, but then the pH was shifted to pH 4.0 the protease activity reduced dramatically. This would be expected from trypsin-like proteases.

From a matched SDS PAGE gel without MAB02, the 29 kDa band was cut from the gel and subjected to in-gel trypsin digestion. In the purified fractions, the 29 kDa band was seen as a distinct protein band that was isolated from the gel. The purified peptides were analyzed by LC-MS/MS. The resulting mass analysis clearly identified the 29 kDa band as the trypsin-like serine protease TSP1 (tre73897, 35% sequence coverage). TSP1 was the first major serine protease identified, thus it was the second protease deleted after *pep1*.

The protease producing the activity around 65 kDa was more difficult to identify due to its low expression level and proximity in size to several highly expressed proteins such as CBHI (tre123989), CBHII (tre72567), and xylanase 4 (tre111849). A lower gel percentage (7%) SDS PAGE gel was used for zymogram and standard SDS PAGE gel and run until the 54 kDa molecular weight marker was at the bottom of the gels to improve the separation. The general region was cut, subjected to in-gel trypsin digestion, and the resulting peptides were analyzed by LC-MS/MS. The peptide analysis showed that the second highest scoring protein was the protease tre51365. The top scoring protein was xylanase4, which was a contaminant in the sample. The tre51365 subtilisin protease, SLP1, was found in 3 independent samples from three separate purifications. In the best scoring sample, 6 peptides were found and sequenced by LC-MS/MS. The sequence coverage was 8%, since the native protease gene codes for 882 amino acids that compose a 93 kDa protease. In gelatin zymography, a weak band at ~90 kDa could be seen along with smearing down to 65 kDa, suggesting that the SLP1 protease itself undergoes proteolysis but retains much of its activity. The SLP1 protease was the second serine protease identified in this study, so it was the third protease removed from the multiple deletion strain.

**Fig 5:**
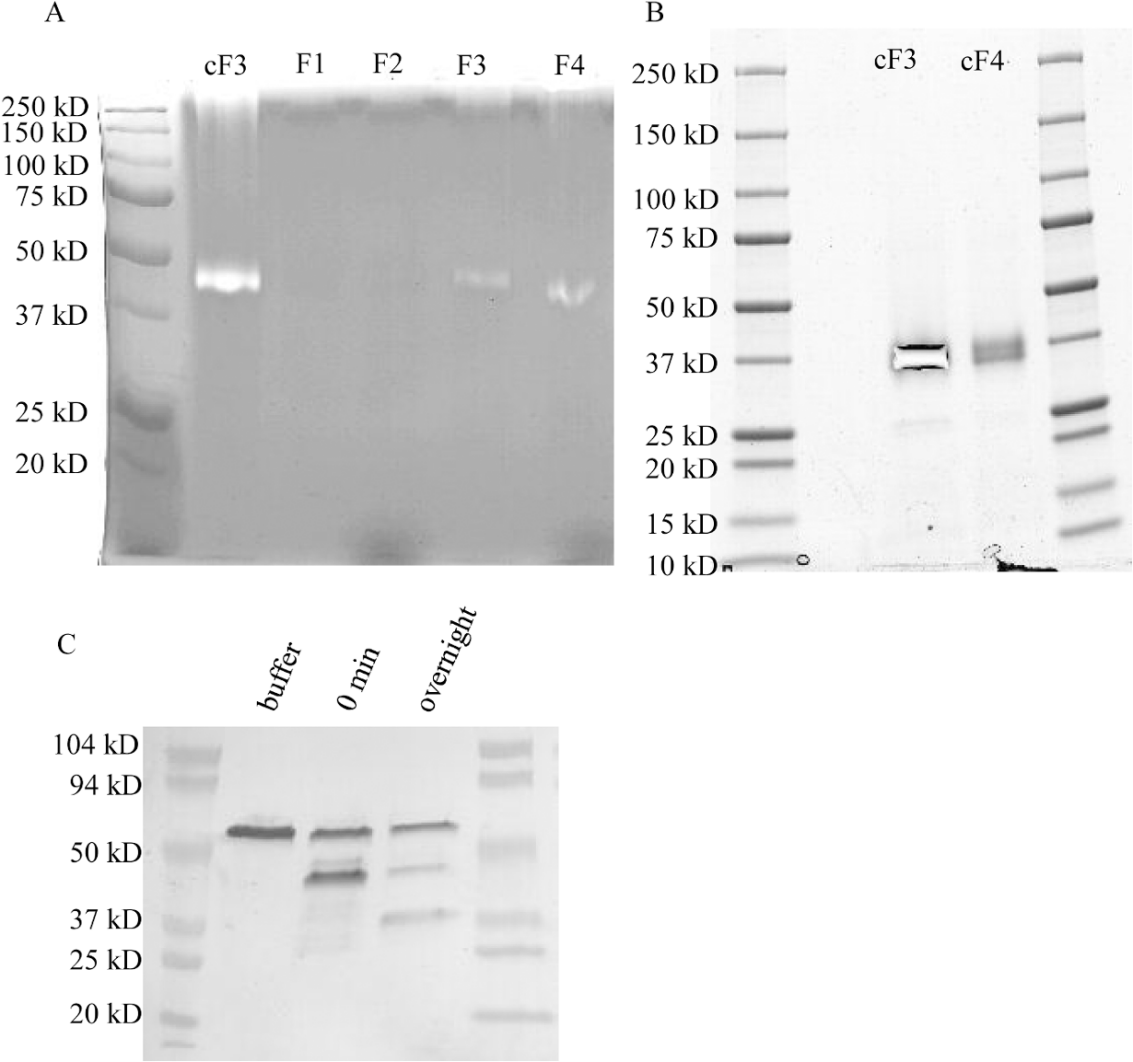
SBTI purified serine proteases degrade antibody. Fig 5A. MAB02 zymogram assay with SBTI affinity purified fractions containing proteases. The major proteolytic activities appear white, where the protease has degraded the MAB02 antibody. The major activity is located at 40 kDa. Fractions 1–4 (F1-F4) and concentrated fraction 3 (cF3) are shown. Fig 5B. SBTI affinity purified fractions containing proteases run on a SDS PAGE gel. The concentrated fractions 3 (cF3) and 4 (cF4) show a doublet around 38 kDa and a weaker band at 26 kDa. Fig 5C. Rituximab heavy chain degradation by SBTI purified proteases at pH 5.5 and 37°C for 18 hours. The purified proteases rapidly degraded the rituximab heavy chain into two major products that run at 38 and 45 kDa. The full length heavy chain runs just over 50 kDa. Immunoblot detection with antihuman IgG heavy chain specific AP conjugated antibody diluted 1:30,000 in TBST.

The soybean trypsin inhibitor, SBTI, effectively stabilized the antibody heavy chain against protease degradation. Therefore, SBTI was able to inhibit proteases that are responsible for cleaving the antibody. Soybean trypsin inhibitor was used to purify proteases from fermentor culture supernatant from the M44 strain. From all the fractions collected, 190 μg of protein was purified using the SBTI affinity column from 20 ml of culture supernatant. The concentrated peak fractions and unconcentrated fractions were loaded on an MAB02 zymogram gel and on a regular SDS PAGE gel for analysis. The results of the zymogram showed that there are two proteolytic activities. The most predominant band was easily visible around 40 kDa (Fig 5A), while the 26 kDa activity was too faint to appear in the figure. In the zymogram gel, darker staining protein bands flanked the white zymogram activity band. Comparing this to concentrated fractions loaded on an SDS PAGE gel, these doublet bands could be seen around 38 kDa (Fig 5B). The PAGE gel was a 4–15% gradient gel and the zymogram gel was 12%, so the relative sizes can be slightly different. On the PAGE gel, a protein band was seen in the area of 26 kDa, which corresponded to the size of the second fainter zymogram activity.

To further analyze the proteolytic activity of the purified protease in concentrated fraction #3, the fraction was tested for its ability to degrade the rituximab antibody heavy chain. The results of mixing rituximab and the SBTI purified proteases were visualized via immunoblot, which showed that the proteases immediately degraded the rituximab heavy chain after mixing and sampling. The full length rituximab heavy chain runs at just over 50 kDa, while the initial degradation product was around 45 kDa (Fig 5C). Additionally, incubation overnight generated an additional product of 38 kDa. These are the same sized degradation products as seen after incubating rituximab with culture supernatant, as seen in Fig 3. The purified proteases studied here are only faster at degrading the heavy chain.

To identify the proteases responsible, slices were cut from the SDS PAGE gel corresponding to observed zymogram activities and digested with trypsin for LC-MS/MS analysis. The top scoring hit from 38–40 kDa region was a galacturonase (tre122780). The second highest hit was the subtilisin-like protease, SLP2 (tre123244). Two peptides from SLP2 were found and sequenced, covering 6% of the entire sequence length. The full length SLP2 protease is 58 kDa, but it is usual that the active protease can be smaller in size. There were also other proteases found in adjacent regions. Analysis of the lower 26 kDa region identified the trypsin serine-like protease TSP1 (tre73897). This corresponded to the faint zymogram activity observed. As described above, this protease was also identified via aminobenzamidine affinity purification.

In addition, the whole SBTI affinity purified fraction was trypsin digested in solution to determine the entire protease content of the sample. Other identified proteases included the tre123865 protease SED1 (60 kDa); the tre77579 protease PEP4 (42 kDa); the tre58698 protease SLP8 (41 kDa); tre122703, metalloprotease (53 kDa); and tre60581, metalloprotease (80 kDa). These proteases appear to bind to the SBTI protein and are inhibited by it.

From the chymostatin affinity column, the whole purified fraction was analyzed for protease content via LC-MS/MS. In the purified fraction the top hit was the subtilisin protease SLP2 (tre123244). It was the only endoprotease found in the fraction. The SLP2 protease was the third major serine protease found in this study. It would have been the fourth protease removed from the multiple deletion strain, but deletion of *slp2* resulted in slower strain growth and sporulation problems. Thus, it was not removed from the multiple deletion strain.

### Triple deletion strain M277

**Fig 6:**
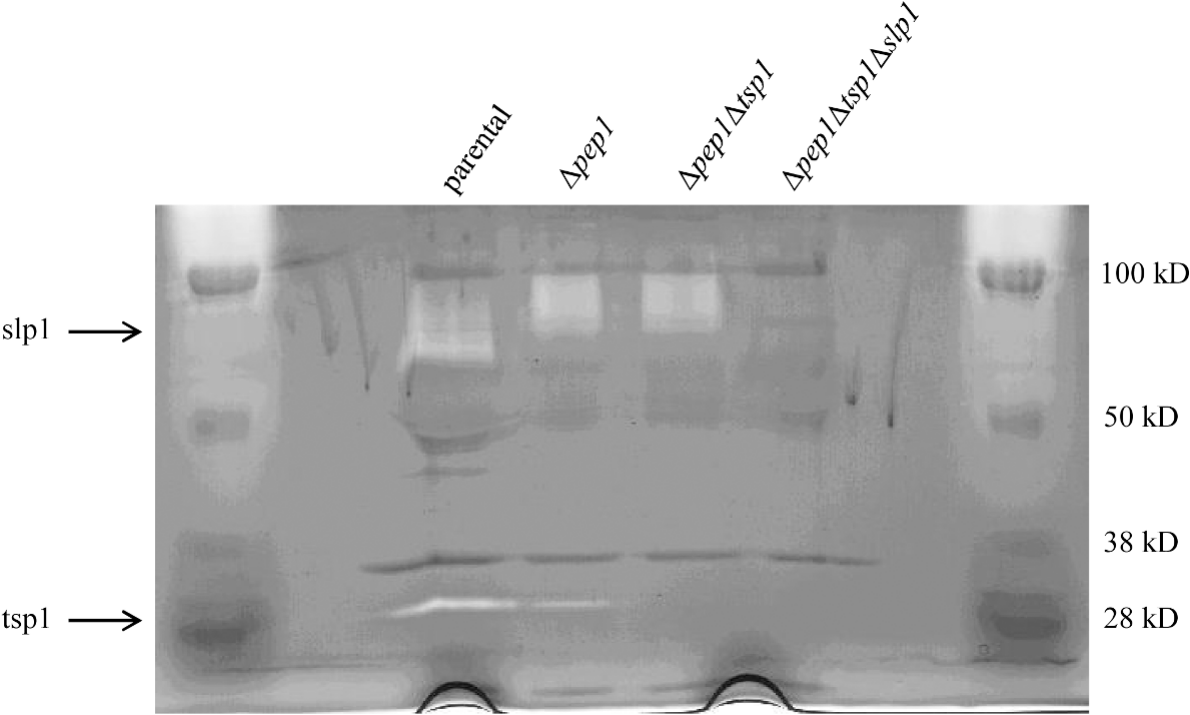
MAB02 zymogram activity of protease deletion strain supernatants. White regions on the blue stained gel indicate an area of protease activity against MAB02. The major activity occurs between 65–90 kDa and a smaller protease activity occurs at 28 kDa. Culture supernatant samples from day 7 were diluted 1:2 in sodium citrate buffer (50 mM, pH 5.5) and 30 μl was loaded into a 12% zymogram SDS PAGE gel containing MAB02. The M124 parental strain, M181 (Δ*pep1*), M219 (Δ*pep1*Δ*tsp1*), and M277 (Δ*pep1*Δ*tsp1*Δ*slp1*) supernatants were assayed.

After identifying TSP1 and SLP1 as abundant proteases having activity against IgG, the protease deletion series was continued from the *pep1* deletion strain by deletion of the *tsp1* and *slp1* genes. A study was conducted to compare the relative protease activity of the single, double, and triple protease deletion strain supernatants. The M124 parental strain, the M181 (Δ1), the double deletion M219 (Δ2), and the triple deletion M277 (Δ3) culture supernatants from day 7 shake flasks were analyzed directly with antibody zymography. Two protease activity regions were observed in control M124 and the M181 (Δ1) supernatant (Fig 6). The most predominant one was between 65–90 kDa due to SLP1 and a fainter activity around 28 kDa originating from TSP1. As expected, the M219 (Δ2) strain no longer produced a zymogram band at 28 kDa. Likewise, the M277 (Δ3) strain did not produce either zymogram activity. These results confirm that the correct proteases were identified and deleted. It also indicated that SLP1 protease, under these conditions, was more active than TSP1. The active size of SLP1 appears to be variable, since it is still active when it is cleaved down to 65 kDa even though its mature size is 90 kDa.

**Fig 7:**
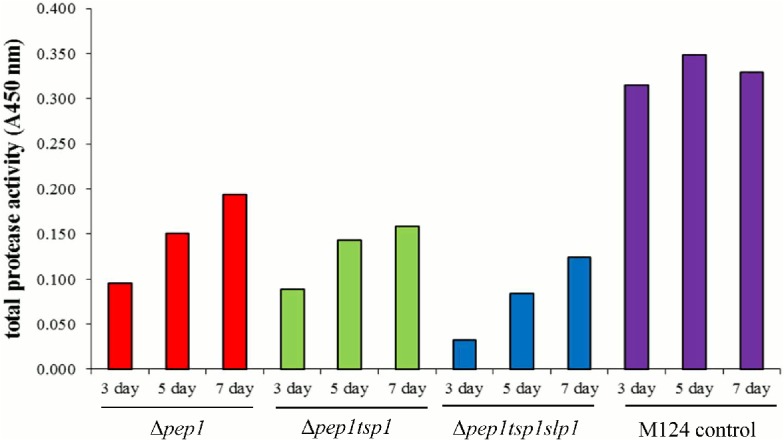
Total protease activity of protease deletion culture supernatants. The relative ability of each culture supernatant to degrade succinylated casein is shown. The supernatants were diluted to 2 mg/ml total protein in 50 mM sodium citrate, pH 5.5 before being assayed. 50 μl of diluted supernatant was loaded into a 96 well plate and 50 μl of succinylated casein was added to begin the reaction. The M181 (Δ1), M219 (Δ2), M277 (Δ3) strain supernatants display less protease activity compared to the M124 control strain.

The total protease activity against casein from these deletion strain supernatant cultures was measured from day 3, day 5, and day 7 samples. The supernatants were diluted to 2 mg/ml total protein before being assayed with succinylated casein. The total protease activity from all the deletion strain supernatants was lower than the M124 strain (Fig 7). The protease activity measured in each strain increased each day and was maximal on day 7. The most productive deletion was Δ*pep1*, followed by Δ*slp1* as the second most useful. The Δ*tsp1* provided the smallest benefit when considering casein degradation. Comparing day 3 data, the Δ*pep1* deletion showed 3.3 times less, the Δ*pep1Δtsp1* deletion 3.6 times less, and the Δ*pep1Δtsp1Δslp1* deletion gave 9.7 times less protease activity compared to the M124 strain supernatant.

**Fig 8:**
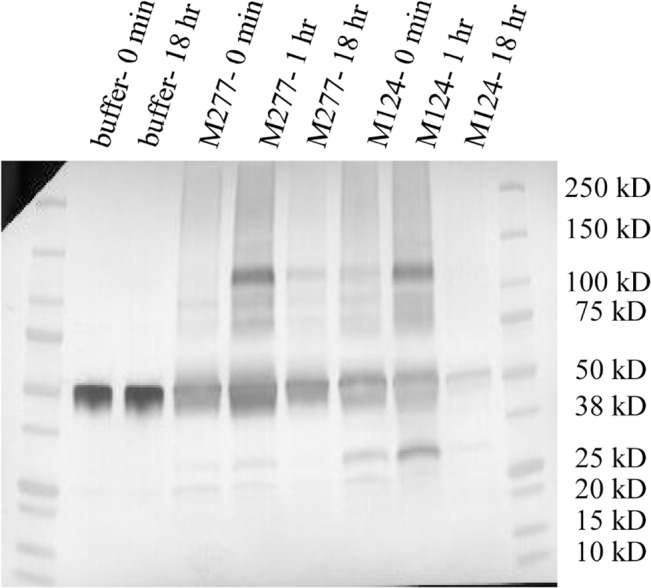
Heavy chain stability improved after removal of three proteases. Immunoblot showing reduced MAB01 heavy chain degradation after incubation with supernatant from strain M277 (Δ3) and control strain M124. Supernatants from day 7 were diluted to 6 mg/ml total protein in pH 5.5 sodium citrate buffer before adding MAB01 antibody (0.05 μg/μl) and incubating at 37°C for 18 hours. The reactions were sampled at zero time, 1 hour, and 18 hours. The 20 μl samples were loaded into a 4–15% SDS PAGE gel and immunoblotted to detect the heavy chain of MAB01 with an anti-heavy chain AP conjugated antibody.

To check stability of antibody heavy chain in the triple protease deletion supernatant the MAB01 antibody was incubated in the M124 parental supernatant and the supernatant from the M277 (Δ3) strain. Looking at the 18 hour incubated samples it was clearly evident that the 50 kDa heavy chain was degraded more in the M124 strain supernatant. The day 7 culture supernatant from M124 nearly degraded all the antibody heavy chain, while there was still a good amount present in the M277 supernatant. With 3 protease deletions, the M277 strain produced MAB01 heavy chain that was significantly stable. On day 5, there was 2.5-fold more heavy chain in the M277 supernatant after overnight incubation. With the day 7 supernatant, there was 4-fold more heavy chain visible (Fig 8). When considering the lower degradation products at 30 and 25 kDa, the initial time points also indicate a faster degradation rate. The sample taken immediately after mixing shows a major 30 kDa degradation product for M124, but only a faint band with the M277 (Δ3) supernatant. As suggested by the inhibitor studies and zymogram studies, the TSP1 and SLP1 are likely the biggest contributors toward the breakdown of the heavy chain.

### Analysis of protease activity in the 4-, 5-, 6-, 7-fold protease deletion strains

The strains M307 (Δ4), M369 (Δ5), M396 (Δ6), and M486 (Δ7) were cultivated in shake flask cultures. The culture supernatants from day 7 were serially diluted in citrate buffer pH 4.5 and assayed for casein degradation. The data from the most diluted supernatant samples was used to estimate a proteolysis rate for each strain. There was a clear reduction in protease activity in all the deletion strains. The deletions of *gap2*, *pep4*, and *pep3* provided reductions of 23%, 35%, and 40%, respectively, compared to their parental strains.

**Fig 9:**
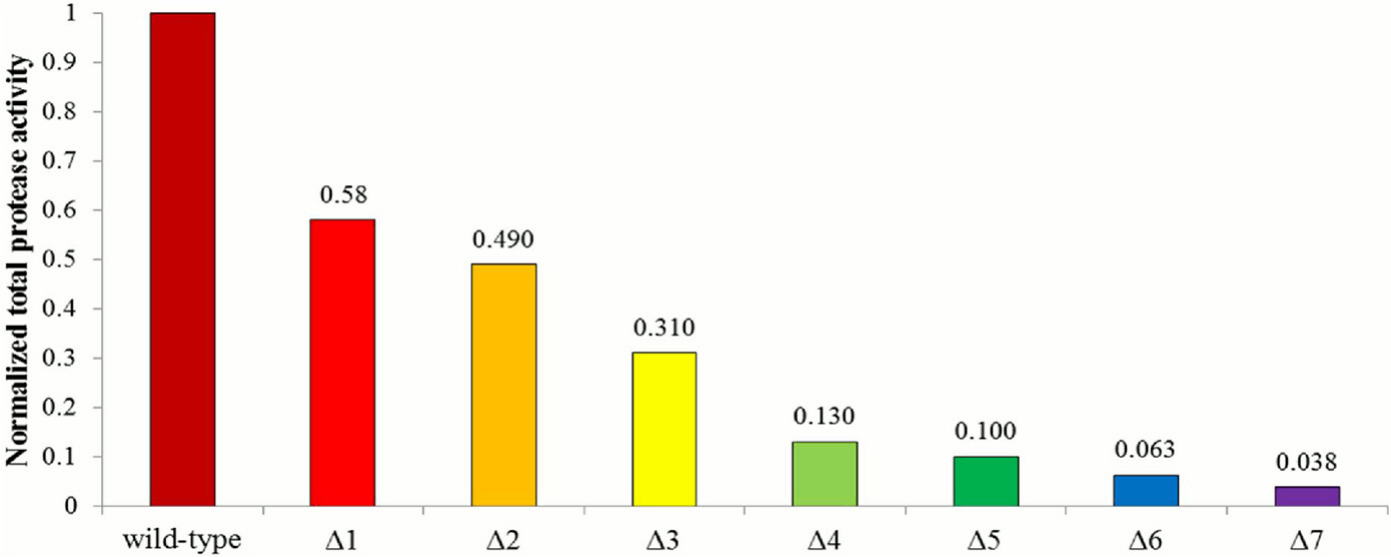
Consecutive deletion of proteases resulted in dramatic reduction in protease activity. Normalized protease activity data from culture supernatants from each of the protease deletion supernatants and the parent strain M124. Protease activity is against a casein substrate. The 6 protease deletion strain has only 6% of the wild type parent strain and the 7 protease deletion strain protease activity went down to 4% based upon casein substrate activity. Wild-type (M124), Δ1 (M181), Δ2 (M219), Δ3 (M277), Δ4 (M307), Δ5 (M369), Δ6 (M396), and Δ7 (M486).

The total protease activity from this analysis was normalized to the previous data with the M181 (Δ1), M219 (Δ2), and M277 (Δ3) multiple protease deletions. This was done because the earliest total protease assays were done with a colorimetric reporter, while the newest assay used fluorescent detection. As the protease activity in the cultures diminished we switched to the more sensitive fluorescent substrate. Yet the substrate, casein, was the same throughout. The results between the two assays correlate well, but the units were different. Thus, a normalized figure was generated. Fig 9 summarizes the total protease activity against casein occurring in the protease deletion strain supernatants compared to M124. The biggest reductions of protease activity were seen after the *pep1* and *gap1* proteases were deleted. The final strain with 7 protease deletions gives only 3.8% of the protease activity of the original strain. This represents a 27-fold reduction in total protease activity against casein.

### Growth and secretion of the multiple protease deletion strains

**Fig 10:**
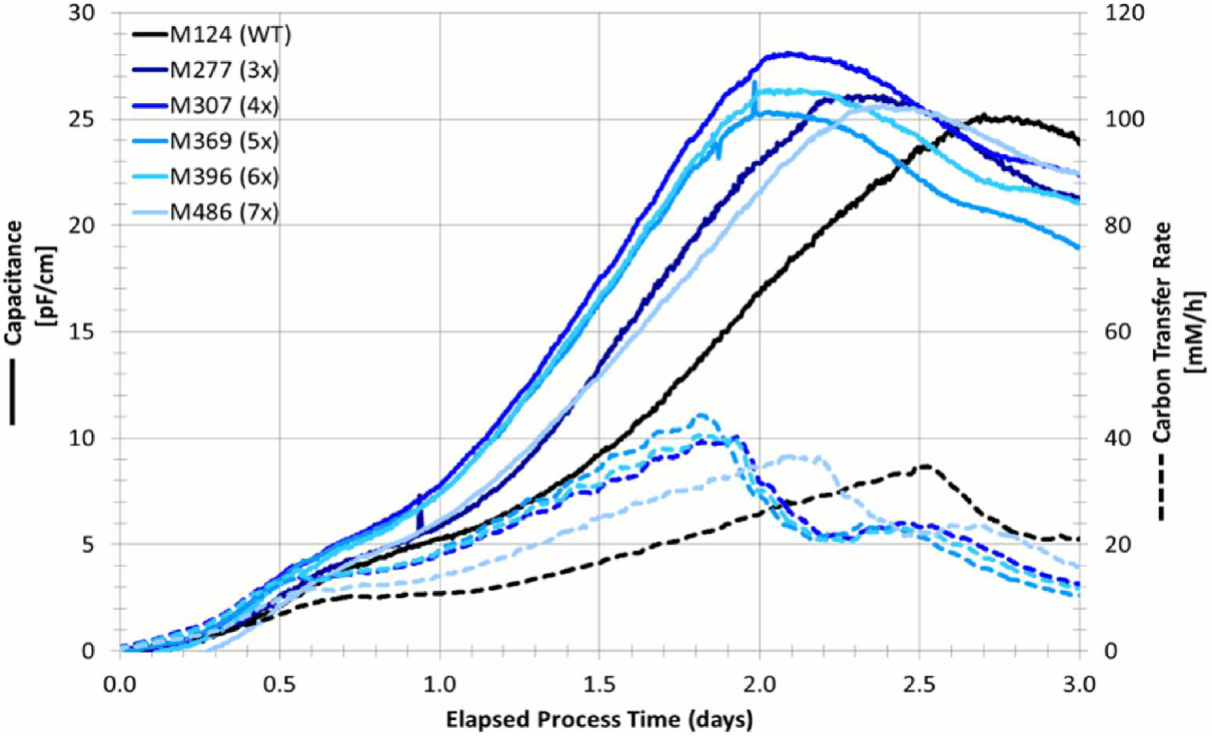
Protease deletion strains grow faster than parental M124 strain. Parallel fermentation data (DASGIP Bioreactor System) showing the growth curves for the M124 strain (wt) and the M277 (Δ3), M307 (Δ4), M369 (Δ5), M396 (Δ6), and M486 (Δ7) multiple deletion strains in complex media. Growth was monitored over 3 days following the Aber Futura biomass signal (capacitance) and CO_2_ transfer rates. (Note: M277 CO_2_ transfer rates are not displayed due to defective off gas monitoring for this single reactor.)

The M486 (Δ7) strain was created after deleting the *pep1*, *tsp1*, *slp1*, *gap1*, *gap2*, *pep4*, and *pep3* proteases from the M124 strain. No negative phenotypes were observed in the deletion strains, but conversely in fermentation culture all strains appeared to grow faster than the original wild-type strain M124 (Fig 10) in complex medium, which is inducing fungal cellulase production. Growth was monitored over 3 days using ABER Futura Biomass Probes (capacitance) and off-gas analysis (CO_2_ transfer rates). Strain M277 (Δ3) grows faster than the zero deletion strain M124 and strains M307 (Δ4), M369 (Δ5) and M396 (Δ6) are ahead of their parental strain M277. Only M486 (Δ7) strain may grow a bit slower than its M396 (Δ6) progenitor strain, but overall M486 propagates faster than M124. Total protein measurements from these cultivation supernatants demonstrated increased protein secretion levels compared to the original strain M124. For instance, the M396 (Δ6) strain produced 22% more total protein than M124 on the last day of culture.

### Therapeutic protein spiking experiments with inhibitors

**Fig 11:**
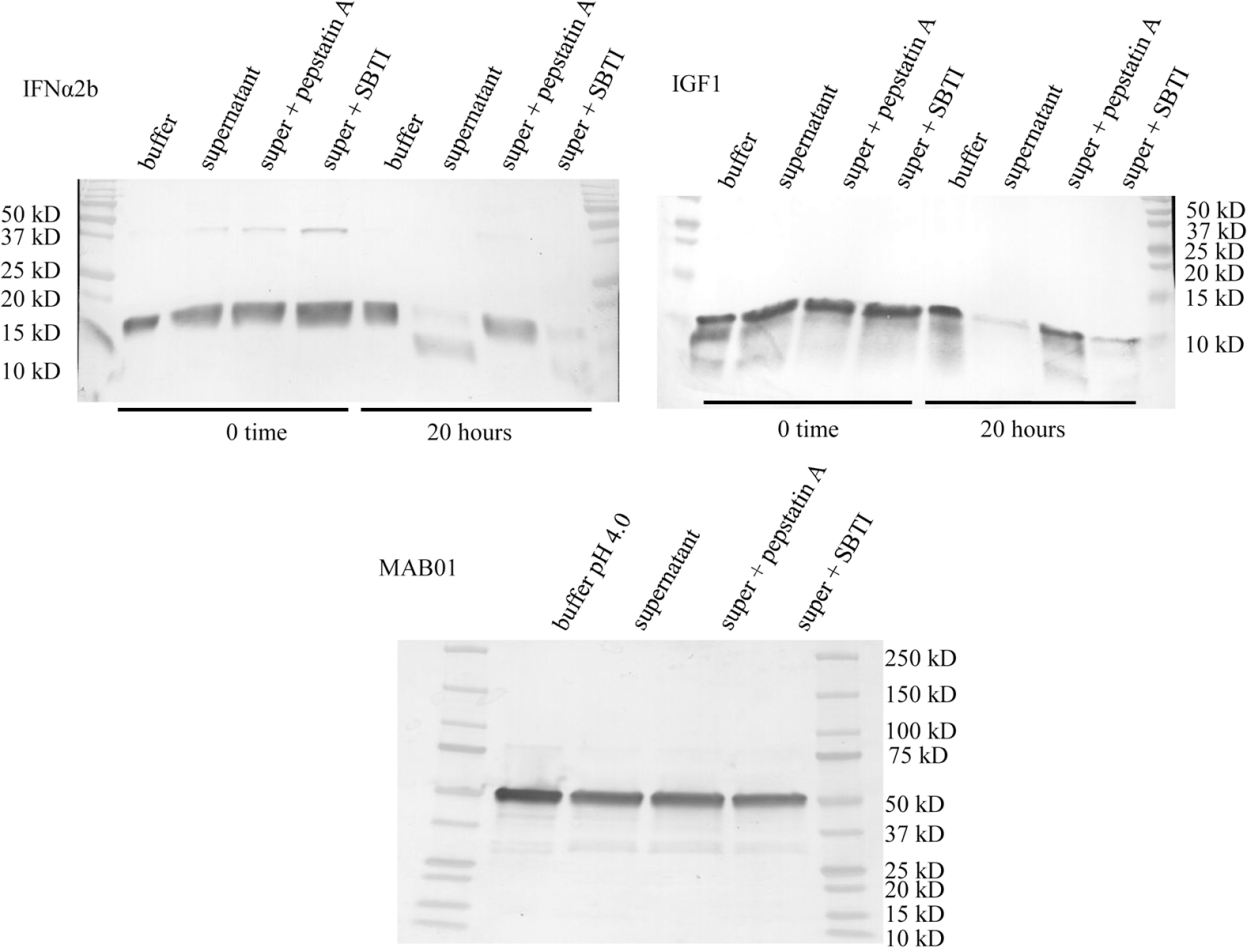
Stability of model therapeutic proteins. Undiluted supernatant from the M396 (Δ6) strain was used at pH 4.2 for spiking in pure model proteins (0.05 μg/μl). 50 mM sodium citrate pH 4.0 spiked with model proteins (0.05 μg/μl) is shown as a buffer control. The spiked supernatant and control were incubated for 20 hours at 37°C. 10 μl of each sample was loaded into 12 or 18% SDS PAGE gels. The IFNα2b ran at 19.4 kDa, the IGF1 ran at 7.5 kDa, and the MAB01 heavy chain ran at 50 kDa.

After deleting 6 proteases from the M124 strain we wanted to compare the relative stability of several model therapeutic proteins in culture supernatant. When incubated for 20 hours in strain M396 (Δ6) supernatant, full length proteins were observed for IFNα2b and IGF1, although the majority appeared to be degraded (Fig 11). There was a predominant degradation product for IFNα2b around 15 kDa. The IFNα2b and IGF1 were remarkably stabilized after treating the supernatant with the aspartic protease inhibitor pepstatin A. This inhibitor blocked the key proteases responsible for the majority of the protease activity. The SBTI provided only a small benefit for product stability. The pH optimum for SBTI is higher than used in the experiment (pH 4.2 vs. optimal pH 8.0) and thus the binding of this inhibitor to its target proteases may not be most efficient.

Pepstatin A effectively inhibits aspartic proteases. It is known from affinity purification studies with pepstatin A that the remaining aspartic proteases in the supernatant are PEP2, PEP3, and PEP5. Therefore, if the remaining 2 or 3 aspartic proteases were deleted the supernatant will be almost free of aspartic protease activity. For production of these model proteins, the aspartic proteases PEP2, PEP3, and PEP5 would be considered major proteases.

This same spiking experiment was done with MAB01 to investigate its stability in the 6 protease deletion supernatant with and without inhibitors (Fig 11). After 20 hours incubation, there was no significant heavy chain degradation. There was no obvious benefit having used inhibitors. The antibody was visibly stable in this pH 4.2 supernatant. The production of MAB01 under acidic conditions would decrease the amount of heavy chain cleavage that would occur.

## Discussion

We have succeeded in reducing the major proteases expressed by *T*. *reesei* in order to tame it into becoming a more suitable system for therapeutic protein production. We have identified 13 proteases that were bound to specific protease inhibitors. These proteases are summarized in Table 2. The major general classes found were aspartic proteases, glutamic proteases, and serine proteases. There were 5 aspartic proteases isolated, but the most important and well expressed one appeared to be PEP1. *Pep1* was deleted first because it was determined to be a predominant protease. Serine proteases were later determined to be most critical for stabilizing antibody heavy chain, thus we began deleting major serine proteases once they were known. The TSP1, SLP1, and SLP2 were the most active serine proteases degrading various antibodies. Once the major serine proteases were removed we continued deleting the most important aspartic and glutamic proteases. Following this general strategy we have sequentially deleted 7 of the most critical proteases from the strain M124.

**Table 2:** Summary of identified proteases.

Gene id	Name	Type	Purification	MW (kDa)	Multiple deletion strain
tre74156	*pep1*	aspartic	pepstatin A	42	yes
tre53961	*pep2*	aspartic	pepstatin A, SIP peptide	42	
tre121133	*pep3*	aspartic	pepstatin A, SIP peptide	49	yes
tre77579	*pep4*	aspartic	SIP peptide, SIP peptide	42	yes
tre81004	*pep5*	aspartic	pepstatin A, SIP peptide	45	
tre69555	*gap1*	glutamic	SIP peptide	26	yes
tre106661	*gap2*	glutamic	-	24	yes
tre73897	*tsp1*	trypsin-like	aminobenzamidine, SBTI	26	yes
tre51365	*slp1*	subtilisin	aminobenzamidine	93	yes
tre123244	*slp2*	subtilisin	SIP peptide, SBTI, chymostatin	58	
tre123865	*sed1*	sedolisin	SBTI	60	
tre58698	*slp8*	subtilisin	SBTI	41	
tre122703	*mep1*	metalloprotease	SBTI	52	
tre60581	*mep7*	metalloprotease	SBTI	80	

Prior to construction of the multiple deletion strain we evaluated some of the protease deletions in a model protein production strain to determine which deletions best improved model protein expression and which were tolerated by the strain. For example, *slp2* deletion was carried out in this way, but even though the *slp2* deletion reduced protease activity it was not eliminated from the multiple deletion strain because of problems with growth and sporulation. The *gap1*, *gap2*, *pep4*, and *pep3* deletions provided the most protease activity reduction amongst the acidic proteases identified and thus were removed from the multiple deletion strain.

When tested in the base strain supernatants the antibody heavy chain was particularly susceptible to proteolytic degradation. Serine proteases appeared to be most responsible for proteolysis. The SBTI and chymostatin inhibitors combined could prevent degradation of the rituximab heavy chain (Fig 3). The inhibitor studies suggest that there are at least two initial cleavage sites in the rituximab heavy chain. The size of the heavy chain fragments indicate that these proteases were not cutting within the hinge region, which is typically a more susceptible region. SBTI prevented the formation of the 38 kDa degradation product and chymostatin was more effective at reducing the 48 kDa product, suggesting that the inhibitors may inhibit different kinds of proteases. In principle, the SBTI inhibitor should work better against trypsin-like proteases like TSP1, while chymostatin would work better against chymotrypsin-like proteases like SLP1 and SLP2.

When the MAB01 was incubated with M124 or supernatant from strain M277 (Δ3) there were heavy chain fragments at 25 and 28 kDa (Fig 8). These suggest cleavage sites within the hinge region of the heavy chain. The stability of the full length heavy chain improved dramatically when incubating in the triple protease deletion strain supernatant. The aspartic protease *pep1*, the trypsin like serine protease *tsp1*, and the subtilisin protease *slp1* were deleted from the strain. This suggests that these three proteases are of major importance for heavy chain stability. The proper identification of TSP1 and SLP1 as MAB02 degrading proteases was clearly demonstrated (Fig 6). The zymogram reactivities previously seen were no longer visible in the strain M277 (Δ3). Based upon additional zymogram reactivity studies SLP2 was also found to degrade the MAB02 antibody (Fig 5A).

The aspartic proteases were not working with the zymogram method, thus no activities were visible. Presumably the aspartic proteases do not refold and retain activity after removal of SDS from the zymogram gel. Studies done with M181 (Δ*pep1*) strain supernatant demonstrated a more stable environment for antibody heavy chains. Additionally, human IgG could be partially stabilized in culture supernatant after treatment with the aspartic protease inhibitor pepstatin. These experiments provide evidence for PEP1 activity upon the antibody. The role of aspartic proteases in antibody degradation was further supported by *Pichia* produced PEP3, which showed pH dependent activity upon the heavy chain (S2 Fig). Likewise the glutamic protease GAP2, produced in *Pichia*, also directly demonstrated proteolytic activity upon the MAB01 heavy chain (S3 Fig). The fragments generated were around 25–28 kDa, indicating they might be hinge cleavage products. Cleavage of antibody heavy chain hinge could be suppressed in *Ogataea minuta* when a yapsin aspartic protease was deleted from the strain [16]. These yapsins are plasma membrane anchored proteases, but their GPI anchors can be released and the proteases can enter the culture supernatant [45]. We did not identify these types of aspartic proteases in this study, but putative gene models for such proteases do exist in the genome and they have been found to be present in the *T*. *reesei* culture supernatant by other investigators [35]. They may play a role in heterologous protein degradation in *T*. *reesei*, but it is unclear how well tolerated the deletions would be. Yapsins are reported to important for maintaining cell wall integrity [46].

The best improvement in antibody stability could be observed when the MAB01 antibody was incubated in the M486 (Δ6) strain supernatant at acidic pH. No protease related degradation products could be observed from the heavy chain. In the M486 (Δ6) strain all the major aspartic and glutamic proteases have been deleted and the lower pH reduced the potential protease activity derived from serine proteases. The M486 (Δ6) strain may work well as a production host for antibodies.

Yet, the 6-fold protease deletion strain supernatant still has protease activity. The other model proteins tested, IGF1 and IFNα2b, were less stable in the supernatant of the M486 (Δ6) strain compared to the antibody. However, considerable amounts of the full length proteins were seen after overnight incubation. There are several other aspartic proteases known and potentially expressed in the supernatant such as PEP5, PEP8, PEP9, PEP11, and PEP12 [35]. One or more of these aspartic proteases may have to be deleted in order to fully stabilize IGF1 and IFNα2b in the supernatant. The remaining serine proteases such as SLP2, SED1, and SLP8 are likely playing a role as well, but the acidic pH may reduce their proteolytic effects. The general protease activity measured from the protease deletion strain series decreased each time we had made a protease deletion. With the M486 (Δ7) strain the casein related protease activity was difficult to measure from the supernatant. There was less than 4% activity remaining compared to the original strain (Fig 9).

Seven protease deletions is currently the highest number reported from *T*. *reesei*. Previously there was a patent application describing deletion of three proteases (*slp1*, *pep1*, and *tsp1*) from *T*. *reesei*, which showed improved storage stability of cellulase enzyme [47]. Ten proteases have been disrupted in the filamentous fungus *A*. *oryzae*, improving production of chymosin and human lysozyme [17]. However, after 10 deletions the total protease activity measured using casein was reduced only around 45%. This may suggest that there are many more proteases in the culture supernatant that would need to be deleted to reduce the casein protease activity further. In contrast, with *T*. *reesei* we have achieved a 96% reduction with only 7 deletions. Protease genes have been surveyed in the *Aspergilli* genomes and there were typically over 300 putative genes per species [48]. In *A*. *oryzae* there are 336 putative genes and 57 of these proteases were secreted, as determined by proteomics. In comparison there were up to 39 secreted *T*. *reesei* proteases [35]. Thus, the protease load in *T*. *reesei* may be somewhat lower.

Interestingly, after deletion of 7 proteases it was observed that the generated protease deletion strains grew better in the fermentor than the M124 parental strain. Most importantly, after multiple selection and marker recycling steps and protease deletions the protein secretion capability of our strain remained intact. Total protein concentrations were measured from the fermentation supernatants from the multiple deletion strains and showed higher levels compared to the M124 strain. For example, the M396 (Δ6) strain produced 22% more total protein. This effect may be due to compensatory mechanisms related to the inability to derive necessary nutrients from protein breakdown. The response may be to secrete higher levels of cellulase enzymes. This has been observed in protease deficient mutants of *A*. *fumigatus*, which secreted higher amounts of polysaccharide degrading enzymes [49]. It is also possible that the native proteases degrade the secreted native proteins to some extent.

In the protease deletion strains we also observed increased growth rate. The first big improvement in growth occurred after the first three protease deletions. The second big improvement occurred after deletion of the *gap*1 glutamic protease. PEP1 and GAP1 are likely to be the two most important acidic proteases secreted by *T*. *reesei*. Out of 7 proteases deleted in the strain, 5 were the major acidic proteases PEP1, PEP4, PEP3, GAP1, and GAP2. As would be expected, our supporting *in vitro* activity studies against model proteins with *Pichia* produced GAP2 and PEP3 suggest pH dependency and higher activity in acidic conditions (S2 and S3 Figs). Possible explanations for the improved growth could be related to the higher amounts of enzymes secreted and improved stability of secreted cellulase enzymes. It has been reported that acid proteases, such as glutamic and aspartic proteases, are able to cleave alpha-galactosidase and cellobiohydrolases. When tested *in vitro* the aspartic proteases were able to cut off the cellulose binding domain from the cellobiohydrolases [30,34]. At pH levels below 3, the protease activity was no longer limited and the entire core enzyme was degraded. The alpha galactosidase II and III were prone to aspartic protease degradation, while alpha galactosidase I and beta glucosidase were more resistant [30]. This activity is not only related to aspartic proteases. The TSP1 protease was found in *T*. *reesei* previously and identified as a major protease degrading the endoglucanase I (Cel7B) [32]. The protease has a neutral pH optimum, so it is not likely to be very active at acidic pH. Based on this line of reasoning we would expect that the protease deletion strains would have a more active set of cellulase enzymes that can more quickly convert sugars and facilitate the strains to grow faster.

The protease reduction in microbial production systems can be done multiple ways such as UV mutagenesis, protease deletions, or deletion of protease regulatory proteins. Our approach to make single deletions of major proteases has been successful so far. We first evaluated many of the protease deletions in a model protein production strain to decide which deletions best improved expression and which were tolerated by the strain. This is an important step to ensure that the most productive improvements can be made without any major side effects. The downside of making multiple deletions with marker recycling is that it is a relatively slow process, particularly when performed in filamentous fungi. However, with the advent of new genome editing tools like CRISPR/Cas9 this process can be accelerated dramatically considering multiple proteases can be deleted each round [50]. Deletion of regulatory factors that control protease expression may be another option to achieve protease activity reduction, but it may be difficult to find regulators that exclusively govern proteases. As protease secretion is intimately integrated with carbon and nitrogen regulation, this concept may have downsides. For example, while *prtT* in *A*. *fumigatus* controls multiple proteases, the deletion of *prtT* decreased expression of genes involved in iron uptake and ergosterol synthesis, but upregulated genes involved in secondary metabolite biosynthesis [49]. Similarly, UV mutagenesis can produce multiple unknown changes in the genome and lead to unintended consequences. Thus, deletion of multiple preselected proteases may be the least harmful approach to improving heterologous protein expression.

The multiple protease deletion strain of *T*. *reesei* generated in the present study should already be a good production strain for production of antibodies and many other recombinant proteins, even for sensitive therapeutic proteins such as IFNα2b and IGF1. Further work will be done to improve the strain, delete additional proteases, optimize fermentation conditions, and demonstrate its capabilities as a therapeutic production system.

## Supporting Information

S1 Fig Plasmid containing deletion construct for *pep1* protease. The deletion construct contains 5′ and 3′ flank sequences and a *pyr4* loopout marker. The deletion construct is flanked by *PmeI* digestion sites to allow removal from the plasmid backbone.
(TIF)

S2 Fig Production of *T*. *reesei* PEP3 in *Pichia pastoris*. Western blot and SDS-PAGE analysis from 10X concentrated PEP3 supernatants, expressed at +30°C for 3 days in MeOH induction (panel A). PEP3 was active against casein at all pHs tested (panel B) and also degraded MAB01 heavy chain (panel C) and IGF (panel D).
(TIF)

S3 Fig Production of *T*. *reesei* GAP2 in *Pichia pastoris*. SDS-PAGE and Western blot (with anti-strep tag antibody) of supernatant of *Pichia* produced *Trichoderma reesei* GAP2 and mock control (panel A). The diluted 1x concentrate was applied to MAB01 for 0 min or 20 hr at pH 4.0–5.5 (panel B) and IGF at pH 4.0 (panel C). GAP2 is most active at pH 4.0 against MAB01 heavy chain (0.05 μg/μl final) and has reduced activity at higher pHs whereas IGF1 (0.05 μg/μl final) was readily degraded at pH 4.0.
(TIF)

S1 Table Primers for generating deletion constructs. (DOCX)

S1 Text Supporting methods and results. (DOCX)
