# Supplementary figures and images for: Enabling Low Cost Biopharmaceuticals: A Systematic Approach to Delete Proteases from a Well-Known Protein Production Host Trichoderma reesei
Source: PLoS One. 2015 Aug 26;10(8):e0134723. doi: 10.1371/journal.pone.0134723 (PMC4550459; doi:10.1371/journal.pone.0134723)

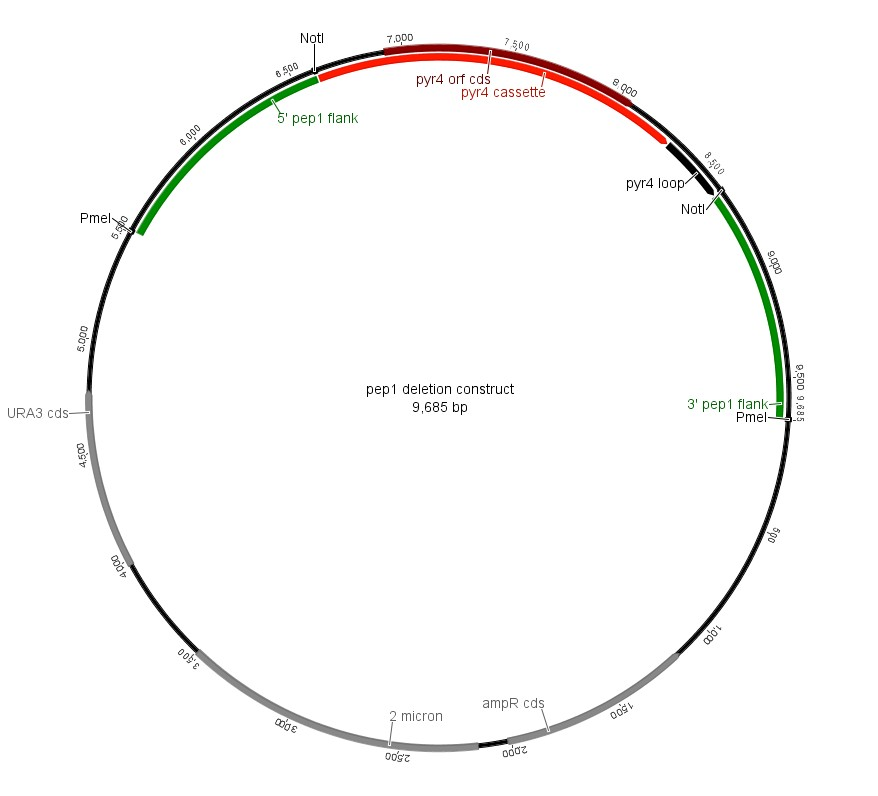

Supplement: S1 Fig — The deletion construct contains 5′ and 3′ flank sequences and a pyr4 loopout marker. The deletion construct is flanked by PmeI digestion sites to allow removal from the plasmid backbone. (TIF) [file pone.0134723.s001.tif]

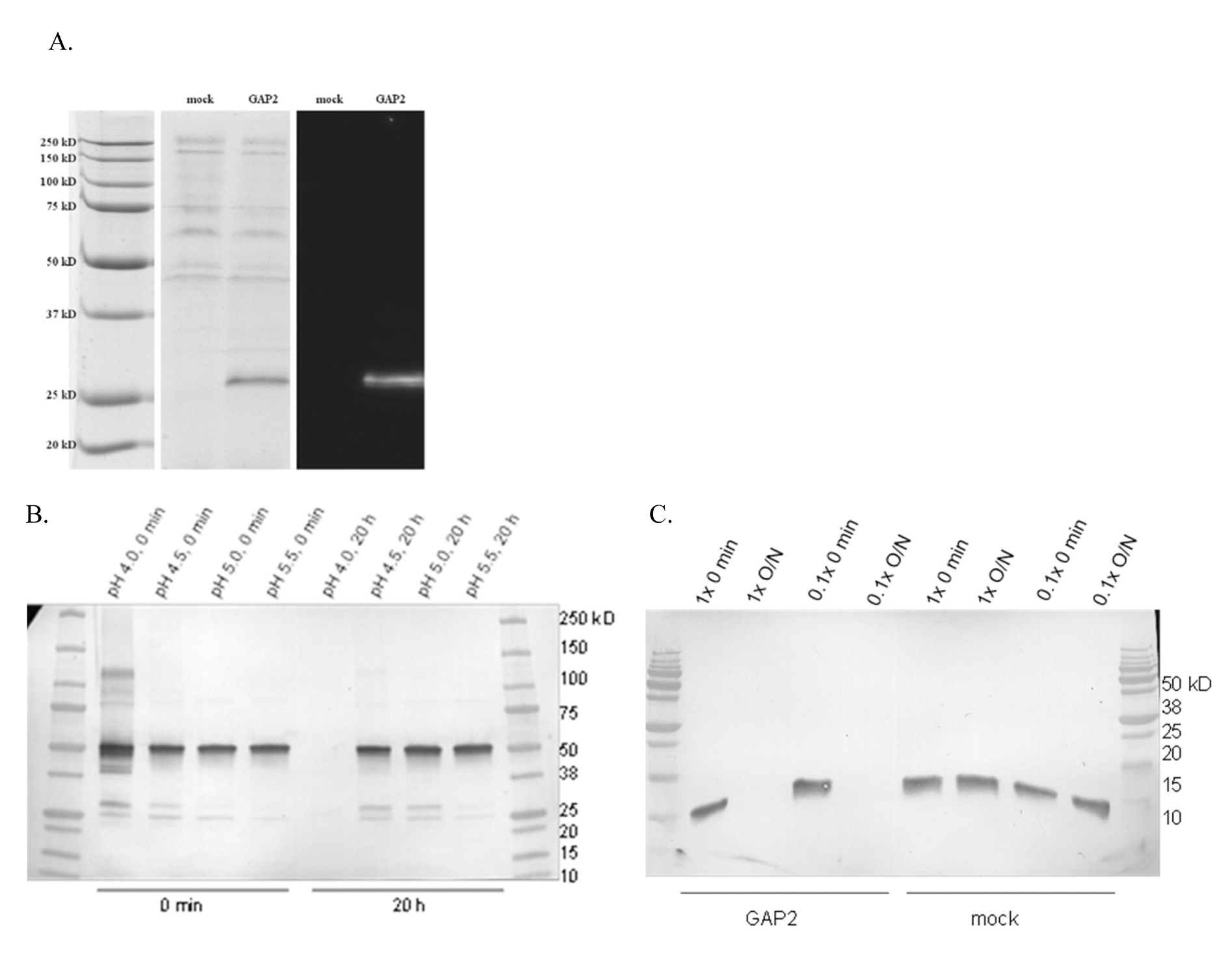

Supplement: S3 Fig — SDS-PAGE and Western blot (with anti-strep tag antibody) of supernatant of Pichia produced Trichoderma reesei GAP2 and mock control (panel A). The diluted 1x concentrate was applied to MAB01 for 0 min or 20 hr at pH 4.0–5.5 (panel B) and IGF at pH 4.0 (panel C). GAP2 is most active at pH 4.0 against MAB01 heavy chain (0.05 μg/μl final) and has reduced activity at higher pHs whereas IGF1 (0.05 μg/μl final) was readily degraded at pH 4.0. (TIF) [file pone.0134723.s003.tif]
