# Supplementary material for: Enabling Low Cost Biopharmaceuticals: A Systematic Approach to Delete Proteases from a Well-Known Protein Production Host Trichoderma reesei
Source: PLoS One. 2015 Aug 26;10(8):e0134723. doi: 10.1371/journal.pone.0134723 (PMC4550459; doi:10.1371/journal.pone.0134723)
Supplement: S1 Table — (DOCX) [file pone.0134723.s004.docx]

**S1 Table.**

| **Primers for generating pep1 deletion plasmids.** | | |
| --- | --- | --- |
| Deletion plasmid pTTv41 for pep1 (TreID74156), vector backbone pRS426 | | |
| Primer | Sequence | |
| 5flankfw | GTAACGCCAGGGTTTTCCCAGTCACGACGGTTTAAACGTATTGCGATGAGCAGCAGA | |
| 5flankrev | ATCCACTTAACGTTACTGAAATCTGGTCTCCTAACCCACCAAG | |
| 3flankfw | CTCCTTCAATATCATCTTCTGTCTGTGAAATGAGGTCCCTTCC | |
| 3flankrev | GCGGATAACAATTTCACACAGGAAACAGCGTTTAAACCAAACGCAGCAGAAACCATA | |
| PTfwd | GATTTCAGTAACGTTAAGTGGATGCGGCCGCGACAGAAGATGATATTGAAG | |
| PTrev | GACAGAAGATGATATTGAAGGAGGCGGCCGCTTAAGTGGATCCCGGTGAC | |
|  |  | |
| Deletion plasmid pTTv71 for pep1 (TreID74156), vector backbone pTTv41 | | |
| Primer | Sequence | |
| T315_pyr4_for | GGTGGGTTAGGAGACCAGATTTCAGTAACGTTAAGTGGATGCGGCCGCCTAGCATCGACTACTGCTGC | |
| T316_pyr4_rev | GCAGCAGTAGTCGATGCTAGGCGCGCCATGCAAAGATACACATCAA | |
| T317_pyr4_loop_for | TTGATGTGTATCTTTGCATGGCGCGCCTAGCATCGACTACTGCTGC | |
| T318_pyr4_loop_rev | AGGGACCTCATTTCACAGACAGAAGATGATATTGAAGGAGGCGGCCGCGGCTGATGAGGCTGAGAGAG | |
|  |  | |
|  |  | |
| **Primers for generating tsp1 deletion plasmids.** | | |
| Deletion plasmid pTTv42 for tsp1 (TreID71322/TreID73897), vector backbone pRS426 | | |
| Primer | Sequence | |
| T303_71322_5f | GTAACGCCAGGGTTTTCCCAGTCACGACGGTTTAAACTGCTGTTGCTGTTTGTTGATG | |
| T304_71322_5r_pt | CCCGTCACCGAGATCTGATCCGTCACCGGGATCCACTTAAGCGGCCGCCTGTGGTGAGATCTCCAGACG | |
| T305_71322_3f_pt | GCCAAGCCCAAAAAGTGCTCCTTCAATATCATCTTCTGTCGCGGCCGCACTGTGCCCAACAATAAGCAG | |
| T306_71322_3r | GCGGATAACAATTTCACACAGGAAACAGCGTTTAAACCCAAGGCGCTGGCTGTTA | |
|  |  | |
| Deletion plasmid pTTv72 for tsp1 (TreID71322/TreID73897), vector backbone pTTv42 | | |
| Primer | Sequence | |
| no new primers, pTTv42 digested with NotI and ligated with pyr4-loopout fragment from pTTv71 | | |
|  |  | |
|  |  | |
| **Primers for generating slp1 deletion plasmid.** | | |
| Deletion plasmid pTTv126 for slp1 (TreID51365), vector backbone pRS426 | | |
| Primer | Sequence | |
| 5flankfw_vect | GTAACGCCAGGGTTTTCCCAGTCACGACGGTTTAAACATCTCGGAGTGATGCTTCCT | |
| slp1_5flankrev_pyr4Prom | GCGCTGGCAACGAGAGCAGAGCAGCAGTAGTCGATGCTAGGCGGCCGCATCAGACGAAACCAGACGAG | |
| slp1_3flankfw_pyr4Term | CAACCAGCCGCAGCCTCAGCCTCTCTCAGCCTCATCAGCCGCGGCCGCGCGAATCGAGTTGATGATTC | |
| 3flankrev_vect | GCGGATAACAATTTCACACAGGAAACAGCGTTTAAACCTGGTTGGGATCTGACCACT | |
| this was combined with the pyr4-loopout fragment from pTTv71 | | |
|  |  | |
|  |  | |
| **Primers for generating gap1 deletion plasmid.** | | |
| Deletion plasmid pTTv117 for gap1 (TreID69555), vector backbone pRS426 | | |
| Primer | | Sequence |
| JJ-045 primer | | GATTAAGTTGGGTAACGCCAGGGTTTTCCCAGTCACGACGGTTTAAACACCTCATGAGGGACTATGG |
| JJ-046 primer | | GCGCTGGCAACGAGAGCAGAGCAGCAGTAGTCGATGCTAGGCGGCCGCCAAGAAGAGGCAGAGGGTAAT |
| JJ-047 primer | | CAACCAGCCGCAGCCTCAGCCTCTCTCAGCCTCATCAGCCGCGGCCGCCTATACATACTGATGATACA |
| JJ-048 primer | | TGGAATTGTGAGCGGATAACAATTTCACACAGGAAACAGCGTTTAAACGCCCCATGTATGGACTCTAC |
| this was combined with the pyr4-loopout fragment from pTTv71 | | |
|  |  | |
|  |  | |
| **Primers for generating gap2 deletion plasmid.** | | |
| Deletion plasmid pTTv145 for gap2 (TreID106661), vector backbone pRS426 | | |
| Primer | Sequence | |
| T101_gap2_5flank_F_pRS426 | GATTAAGTTGGGTAACGCCAGGGTTTTCCCAGTCACGACGGTTTAAACGCTACTACGCGAGCAAGTG | |
| T102_gap2_5flank_R_pyr4 | GGAACTGTCGGCGATTGGGAGAATTTCGTGCGATCGCGGCGGCCGCCGGATGAAGATGTGCAGTTG | |
| T103gap2-loop_F_pyr4 | AGGGAACATATCACCCTCGGGCATTTTTCATTTGGTAGGCGGCCGCTAAGATATCTTCAAGCTTATGCG | |
| T104gap2-loop_R | CGGATGAAGATGTGCAGTTG | |
| T105gap2_3flank_F_loop | TGTCTCACTTCCACCCATCTCAACTGCACATCTTCATCCGAGCAACAACATGAGGTTCGAA | |
| T106_gap2_3flank_R_pRS426 | CCTATGTTGTGTGGAATTGTGAGCGGATAACAATTTCACAGTTTAAACACAACGCATGTCCAGCTTTTG | |
|  |  | |
|  |  | |
| **Primers for generating pep4 deletion plasmids.** | | |
| Deletion plasmid pTTv43 for pep4 (TreID77579), vector backbone pRS426 | | |
| Primer | Sequence | |
| T298_77579_5f | GTAACGCCAGGGTTTTCCCAGTCACGACGGTTTAAACTCAGGTCAACCACCGAGGAC | |
| T299_77579_5r_pt | CCCGTCACCGAGATCTGATCCGTCACCGGGATCCACTTAAGCGGCCGCTGAATGGGATGGTTCGATTG | |
| T300_77579_3f_pt | GCCAAGCCCAAAAAGTGCTCCTTCAATATCATCTTCTGTCGCGGCCGCAGGTAGACGCTTTGCGAGTG | |
| T301_77579_3r | GCGGATAACAATTTCACACAGGAAACAGCGTTTAAACTGAACTGACGCGGACTGA | |
|  |  | |
| Deletion plasmid pTTv73 for pep4 (TreID77579), vector backbone pTTv43 | | |
| Primer | Sequence | |
| no new primers, pTTv43 digested with NotI and ligated with pyr4-loopout fragment from pTTv71 | | |
|  |  | |
| Deletion plasmid pTTv181 for pep4 (TreID77579), vector backbone pTTv73 | | |
| Primer | Sequence | |
| T209_pyr4_f_recpep4_5f | AAGTTCCCTTCCTCTGGCAGCAATCGAACCATCCCATTCAGCGGCCGCCTAGCATCGACTACTGCTGC | |
| T210_pyr4_r | CATGCAAAGATACACATCAA | |
| T211_pep4_loop_f_recpyr4 | TGATTGTACCCCAGCTGCGATTGATGTGTATCTTTGCATGGCGGCCGCTCAATGTTGACTGCCCCAGG | |
| T212_pep4_loop_r_recpep4_3f | GCACTTCTTAGATACACACACACTCGCAAAGCGTCTACCTGGCGCGCCTGAATGGGATGGTTCGATTG | |
|  |  | |
|  |  | |
| **Primers for generating pep3 deletion plasmids.** | | |
| Deletion plasmid pTTv188 for pep3 (TreID121133), vector backbone pRS426 | | |
| Primer | Sequence | |
| T346_pep3_5f_for | GGTAACGCCAGGGTTTTCCCAGTCACGACGGTTTAAACGTCGAGCCCCCTGGACACCT | |
| T347_pep3_5f_rev | GCGCTGGCAACGAGAGCAGAGCAGCAGTAGTCGATGCTAGGCGGCCGCCATCGCCGTCGCGGACATGA | |
| T348_pep3_loop_for | TGATTGTACCCCAGCTGCGATTGATGTGTATCTTTGCATGGCGGCCGCTCGACGTTGTATCTGCACTC | |
| T349_pep3_loop_rev | GTACGTTCTGATTGCCAACTACGGACCAGACCAGGGCTCCGGCGCGCCCATCGCCGTCGCGGACATGA | |
| T350_pep3_3f_for | GGAGCCCTGGTCTGGTCCGT | |
| T351_pep3_3f_rev | AGCGGATAACAATTTCACACAGGAAACAGCGTTTAAACACGCGCTTCAACATGCCCCA | |
|  |  | |
|  |  | |
| **Primers for generating Pichia expression vector pTTg47** | | |
| Expression of gap2 (Tre ID106661) in pBLARG-SX vector with endogenous signal peptide and with C-terminal strep-tag | | |
| Primer | Sequence | |
| GP105 | ACTAATTATTCGAAACGATGAAGACCCTCGCTTTCGCCACAAACG | |
| GP111 | CTCGAGGTACCCTACTTCTCGAATTGTGGGTGGGACCATTCATACTCAACAGTCACAGTG | |
