# Supplementary material for: Enabling Low Cost Biopharmaceuticals: A Systematic Approach to Delete Proteases from a Well-Known Protein Production Host Trichoderma reesei
Source: PLoS One. 2015 Aug 26;10(8):e0134723. doi: 10.1371/journal.pone.0134723 (PMC4550459; doi:10.1371/journal.pone.0134723)
Supplement: S1 Text — (DOCX) [file pone.0134723.s005.docx]

**Supporting information**

**Production of *Trichoderma reesei* PEP3 and GAP2 in *Pichia pastoris* and activity tests**

*Pichia pastoris* strain GS190 was purchased from Biogrammatics, Inc. (Carlsbad, CA). *Pichia pastoris* codon optimized sequence encoding full-length *T. reesei* PEP3 (tre121133) with C-terminal strep tag II (SA-WSHPQFEK) was cloned into EcoRI/KpnI sites in vector pBLARG-SX together with N-terminal fusion of *S. cerevisiae* α-mating secretion carrier (vector pTTg37).

Vector pTTg37 was verified by sequencing and transformed into *P. pastoris* GS190 strain by electroporation. Positive clones were selected by colony PCR screening from SC-arg plates and three positive transformants from both strains were cultivated for PEP3 expression, at +16°C or +30°C for 1 day in BMGY medium and for 3 days in BMMY medium.

After cultivation at +16°C or +30°C supernatants were concentrated (from 40 ml) by ultrafiltration to 10x by using Amicon ultra-4 centrifugal tubes (MWCO 30 kDa) and analyzed together with cell pellets in Western blot. A strep tagged *Pichia* produced *T. reesei* enzyme served as a positive control and BSA standards were used to estimate concentration of PEP3. IBA Strep Mab Classic (dilution 1:5000) was used for the primary antibody and after washing of the membrane and another incubation with a 1:100 dilution of Santa Cruz Goat anti-mouse IgG with HPR conjugate followed. This conjugate was detected using Millipore HRP detection kit as recommended by the manufacturer and the signals were visualized using Fujifilm LAS 4000 image reader. The diluted 10× concentrates were then used for protease activity studies at pH 4.0-5.5 and concentrates of *P. pastoris* mock strain GY3 (*i.e.* GS190 harboring empty pBLARG-SX) grown under the same conditions were used as negative controls for comparison.

The proteases were produced in *Pichia* supernatant and used to test MAB01 antibody and IGF-1 degradation. The 10x concentrated supernatants were diluted to 1x in 50 mM citrate buffer at pH 4.5 or pH 5.5. To these diluted supernatants MAB01 (0.05 µg/µl final) or native IGF-1 (0.15 µg/µl final) were added and incubated overnight at 37°C. Samples were taken and analyzed by immunoblotting.

The general protease activity upon fluorescent casein substrate was measured from the diluted substrates at these different pHs using the EnzCheck protease assay kit (Molecular probes, #E6638, green fluorescent casein substrate). The working stock solution was prepared by diluting the stock to 10 µg/ml in 50 mM sodium citrate, pH 4.5 or pH 5.5. The purified protease fractions were diluted with sodium citrate buffer. 100 µl of the diluted substrate was combined with the diluted protease fractions in a 96 well sample plate. The plate was then covered and kept at 37°C for one to three hours. Fluorescence readings were taken at one, two, and three hours with a Varioskan fluorescent plate reader (Thermo Scientific) using 485 nm excitation and 530 nm emission.

*T. reesei* GAP2 (tre106661) was cloned by PCR with primers GP105 and GP111 (Supporting table S1) from genomic DNA extracted from *T. reesei* mycelia with Qiagen genomic DNA extraction kit. Amplified sequence with C-terminal strep tag II introduced with GP111 was cloned to *Pichia* expression vector pBLARG-SX with BstBI/KpnI resulting vector pTTg47. After sequencing pTTg47 was linearized by digestion with AvrII restriction enzyme and transformed to *Pichia pastoris* strain GS190 to yield strain GY31. Positive transformants were initially selected by arginine depletion and then screened by colony PCR. Two positive clones, cl3 and cl11 were selected for GAP2 expression in 40 ml volume on BMGY and BMMY (MeOH induction) at +28ºC. After two days on MeOH induction, supernatants were collected, concentrated as 10x and subjected to SDS-PAGE and Western blot analysis with anti-strep tag antibody.

The 10x concentrate was diluted to 1x in sodium citrate buffer adjusted to pH 4.0, 4.5, 5.0, or 5.5. MAB01 or IGF were spiked into the diluted GAP2 supernatant or mock control supernatant so that the final concentration of the protein was 0.05 μg/μl. Samples were taken after 20 h and analysed via immunoblotting with anti-IgG heavy chain antibody AP conjugate (Sigma #A3188; dilution 1:30000 in TBST) or anti-IGF antibody (1:2000 in TBST) using anti-IgG AP conjugated secondary antibody (1:5000 in TBST).

**Results**

**Production of PEP3 and GAP2 proteases in *Pichia***

It was difficult to affinity purify separate aspartic proteases due to their close sequence homology. Therefore, to directly observe their proteolytic effects on model proteins selected proteases were produced heterologously in *Pichia pastoris* and their enzymatic activity studied in diluted supernatant concentrates. We were particularly interested in finding out where the aspartic and glutamic proteases cleaved the antibody heavy chain.

The first methionine of the GAP2 sequence reported at the Joint Genome Institute database (http://genome.jgi-psf.org/Trire2/Trire2.home.html) appeared to be incorrect one as another Met was found in frame 5’ upstream of the genomic sequence of *T. reesei* during cloning and this extended new sequence also harboured a proper signal peptide.

On the other hand, during cloning of PEP3 no evident endogenous signal peptide was found for the ER secretion or any other cellular location using SignalP 3.0 and TargetP 1.1 server. BLAST search retrieved proteins with similar N-terminus supporting the fact that N-terminus of the PEP3 most probably is the correct one and it contains an atypical targeting peptide or PEP3 is an intracellular protease. For *Pichia* expression the matα secretion carrier was fused to the N-terminus to increase likelihood of secretion into the supernatant if the endogenous *Trichoderma* PEP3 N-terminus would not target PEP3 into the ER.

GAP2 (expressed with endogenous signal peptide) and PEP3 (with matα secretion carrier) were both secreted into the *Pichia* supernatant with a level of tens of micrograms of heterologously produced protein per milliliter (Figs. S2 and S3). Hardly any GAP2 or PEP3 protein was detectable in the cell pellets.

Calculated molecular mass for mature GAP2 polypeptide is 25.3 kDa and for mature PEP3 49.4 kDa which correspond to the apparent molecular masses detected in the SDS-PAGE and suggesting that in both proteases endogenous signal peptide or matα secretion carrier were cleaved. Whether one putative N-glycosylation site of the mature GAP2 or PEP3 is glycosylated remains to be determined.

**Activity of PEP3 and GAP2**

The hydrolytic activities of PEP3 and GAP2 were tested in diluted pH adjusted supernatant concentrates. The supernatants, in contrast to purified proteins, were used because initial attempts to isolate a *Pichia* produced *T. reesei* protease by using anti-strep tag column produced fairly low amounts of the purified active protease, necessitating purification optimisation for each of the proteases produced (altogether 20 proteases were expressed in *Pichia*). Casein and therapeutic proteins were spiked into the pH adjusted diluted concentrate solutions at pHs 4.0-5.5 which correspond to culture conditions *Trichoderma* is subjected when producing therapeutic proteins. Finally samples were subjected to SDS-PAGE/Western blot to visualize any degradation products.

PEP3 activity was tested against casein, MAB01, and IGF and showed activity at all pHs tested (Fig. S2). PEP3 was most active against the MAB01 heavy chain at pH 4.5, compared to pH 5.5. This is well expected as aspartic proteases are typically more active under acidic conditions. The more labile IGF1 protein was quickly degraded when in the presence of PEP3.

GAP2 activity was tested on MAB01 and IGF, which were spiked in the diluted concentrates *in vitro*. GAP2 degraded all the tested proteins in pH 4.0 but the activity diminished in pHs 4.5-5.5, whereas in mock supernatants no degradation products were seen (Fig. S3). When GAP2 supernatant was incubated with MAB01 antibody the products of 28 and 25 kDa were seen in pHs 4.5-5.5, which are likely hinge cleavage products (Fig. S3B).
